# Theoretical and Experimental Sets of Choice Anode/Cathode Architectonics for High-Performance Full-Scale LIB Built-up Models

**DOI:** 10.1007/s40820-019-0315-8

**Published:** 2019-10-10

**Authors:** H. Khalifa, S. A. El-Safty, A. Reda, M. A. Shenashen, M. M. Selim, A. Elmarakbi, H. A. Metawa

**Affiliations:** 1 National Institute for Materials Science (NIMS), Sengen 1-2-1, Tsukuba, Ibaraki 305-0047 Japan; 2 Department of Mathematics, Al-Aflaj College of Science and Human Studies, Prince Sattam Bin Abdulaziz University, Al-Aflaj, 710-11912 Saudi Arabia; 3 Department of Mechanical and Construction Engineering, Faculty of Engineering and Environment, Northumbria University, Newcastle upon Tyne, NE1 8ST UK; 4 Department of Physics, Faculty of Science, Damanhur University, Damanhur, Egypt

**Keywords:** Lithium-ion battery, 3D super-scalable hierarchal anode/cathode models, Density functional theory, Anode/cathode architectonics, Electric vehicle applications

## Abstract

To control the power hierarchy design of lithium-ion battery (LIB) built-up sets for electric vehicles (EVs), we offer intensive theoretical and experimental sets of choice anode/cathode architectonics that can be modulated in full-scale LIB built-up models. As primary structural tectonics, heterogeneous composite superstructures of full-cell-LIB (anode//cathode) electrodes were designed in closely packed flower agave rosettes TiO_2_@C (FRTO@C anode) and vertical-star-tower LiFePO_4_@C (VST@C cathode) building blocks to regulate the electron/ion movement in the three-dimensional axes and orientation pathways. The superpower hierarchy surfaces and multi-directional orientation components may create isosurface potential electrodes with mobile electron movements, in-to-out interplay electron dominances, and electron/charge cloud distributions. This study is the first to evaluate the hotkeys of choice anode/cathode architectonics to assemble different LIB–electrode platforms with high-mobility electron/ion flows and high-performance capacity functionalities. Density functional theory calculation revealed that the FRTO@C anode and VST-(i)@C cathode architectonics are a superior choice for the configuration of full-scale LIB built-up models. The integrated FRTO@C//VST-(i)@C full-scale LIB retains a huge discharge capacity (~ 94.2%), an average Coulombic efficiency of 99.85% after 2000 cycles at 1 C, and a high energy density of 127 Wh kg^−1^, thereby satisfying scale-up commercial EV requirements.

**Electronic supplementary material:**

The online version of this article (10.1007/s40820-019-0315-8) contains supplementary material, which is available to authorized users.

## Highlights

- Modulation of 3D super-scalable hierarchal anode/cathode models as choice architectonics into a full-scale LIB design.
- The scalable architectures dynamically provide effective diffusion gateways to guarantee excellent specific LIB performance.
- The DFT theoretical surface–surface electronic and charge map analyses confirmed the superiority of choice anode/cathode architectonics in full-scale LIB built-up models.

**Electronic supplementary material:**

The online version of this article (10.1007/s40820-019-0315-8) contains supplementary material, which is available to authorized users.

## Graphical abstract

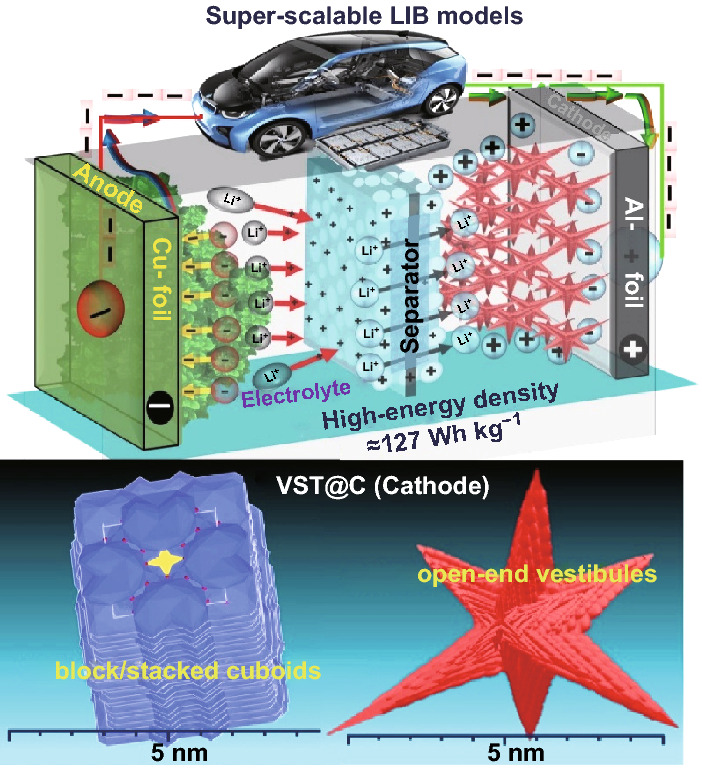

## Introduction

In response to the growing demand for an assortment of high-energy storage systems, energy sources, and environmentally clean and sustainable energy, rechargeable lithium-ion batteries (LIBs) have become predominant electrochemical storage materials for modern transport systems and electronic devices, such as laptops, electronic gadgets, camcorders, and smartphones. Although LIB built-up sets are of particular interests for EVs, LIBs still have some limitations associated with their economic cost and inherent risks [1–4]. Therefore, numerous attempts have been made to improve the performance of rechargeable LIBs and increase their energy density, rates, and life cycle. These limitations have also necessitated the efficient utilization of green energy to meet the requirements of future renewable energy storage systems and promote their use in cutting-edge zero-emission transportation applications, such as plug-in hybrid (PHEVs) and fully electric vehicles (EVs) [5–8]. Since then, the growth of the international vehicle market has been stunted on account of the expense-to-range ratio. Therefore, sustainable hybrid electrode materials must be manufactured with prominent electrochemical functionalities.

Many studies have focused on discovering cathode materials with unique features, such as excellent structural stability, high safety, environment friendliness, and low cost, that can replace hazardous cathode materials, including LiCoO_2_, LiNiO_2_, or LiMn_2_O_4_. Fabrication control of polyanion-type (ZO_4_^y−^: Z = P, S, Si, As, Mo, W) electrode materials with unique features is still challenging. Among these positive electrode composites, phosphor, an olivine-structured LiXPO_4_ (X = Fe, Mn, Co, and Ni) with a theoretical capacity of approximately 170 mAh g^−1^, has received much interest as an excellent cathode candidate for LIBs [9, 10]. The electrochemical behavior of LiXPO_4_ was first studied by Padhi et al. [9]. LiFePO_4_ (LFPO) and LiMnPO_4_ (LMPO) were identified as the best candidates for new-generation rechargeable LIBs [9, 11]. These materials have similar stable cyclability and rate capability induced by their poor electronic and ionic conductivities, which can be overcome by synthesizing the hierarchical multi-diffusive materials of nanosized particles, applying coatings, and doping highly conductive materials, such as graphite or carbon [11, 12]. However, these substances also showed a critical disparity. For instance, LFPO has broader commercial application compared with LMPO given its cycling stability, while LMPO shows some performance restrictions due to the powerless kinetics of electrons and the movement of Li^+^ ions. Moreover, a Jahn–Teller distortion of anisotropy can be observed in Mn^3+^ sites and interface strain results from the apparent discrepancy in volume between LiMnPO_4_ and MnPO_4_ [11–14]. LFPO also has several merits, such as its outstanding structure stability, low material cost, non-toxicity, high theoretical capacitance, and thermally stable behavior at high temperatures. Therefore, LFPO is identified as one of the best cathode candidates for rechargeable LIBs [15, 16]. However, LFPO cathodes have poor electronic and ionic conductivities, poor rate capability, sluggish electrode kinetics, Li^+^ ion transport, Li^+^ ion diffusion at the LiFePO_4_/FePO_4_ interface, and low tap density [17–19]. These problems hinder the large-scale use of LFPO in zero-emission transportation applications, such as PHEVs and EVs. To solve this problem, previous studies have enhanced the electrochemical performance of LFPO by improving its electric conductivity and accelerated the Li^+^ ion diffusion by reducing the particle size, inserting conductive additives, doping with super-valence metal ions, synthesizing hierarchically structured materials with multi-diffusive sites, and coating the LFPO composite with carbon nanoparticles to form an LFPO@C composite [20–27]. The hierarchical structures of LFPO have been recently studied for electron enhancement/Li^+^ ion transportation enhancement with a high load reversible capacity and excellent rate capability [28–30]. LFPO crystal orientation also plays an important role in improving Li^+^ ion kinetics during the lithiation/delithiation process, which in turn is crucial in improving the electrochemical performance of LIBs [28, 31]. Therefore, additional effort must be devoted to finding the appropriate controlled modulation for synthesizing LFPO hierarchical meso-/macro-structured hybrid electrode materials with a desirable crystalline orientation to improve electronic/ionic conductivities, rate capability, and ionic diffusion at the interface. Meanwhile, nano-transitional metal oxides have been widely reported as promising anode materials for LIBs as these materials were studied for the first time by Tarascon et al. [32]. Among these nano-transitional oxides, titanium dioxide TiO_2_ (TO) is one of the most attractive candidates owing to its high safety performance, low price, non-toxicity, pollutant-free characteristics, eco-friendliness, low polarization, and good cyclic stability and reversibility [33, 34].

**Scheme 1:**
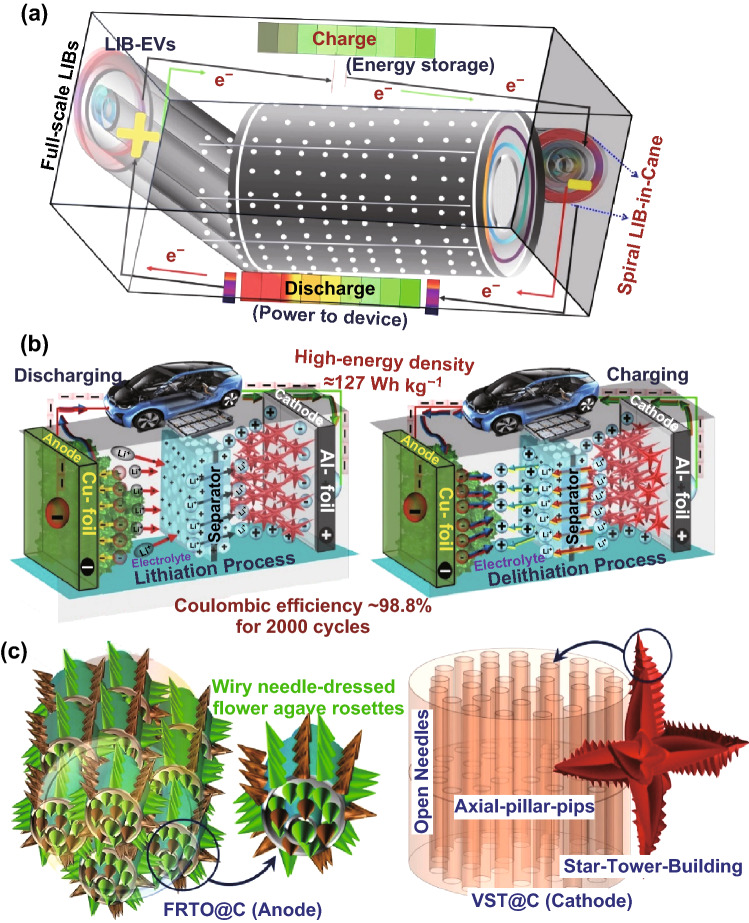
Fabrication of the 18650 cylindrical design of a Li rechargeable battery LIB using power hierarchy built-up sets of negative FRTO@C anode//positive VST@C cathode electrodes for a long-term lithiation/delithiation cycling process (**a**–**c**) for electric vehicles (LIB-EVs). The 3D superstructures of the anode/cathode electrodes of the closely packed flower agave rosettes (FRTO) and in vertical-star-tower (VST) building blocks configured in the full-scale LIBs (**c**). The super structure morphology and chemical composition of the anode/cathode hierarchy play an important role in the LIB full cell design and cycling process (**a**, **b**) for next-generation electric vehicles (LIB-EVs)

To enhance the electrochemical behavior of power hierarchy LIB built-up sets, we offer intensive theoretical and experimental sets of choice anode/cathode architectonics to be modulated in full-scale LIB built-up models. As a mode of choice anode/cathode architectonics in LIB design, novel 3D modulated LIB superstructural models were built based on a LFPO@C vertical-star-tower building block (VST@C cathode)//TiO_2_@C flower agave rosettes (FRTO@C anode) nano-architecture building to guarantee their outstanding electrochemical performance and excellent rate discharge capacities over a potential range of 2.0 to 4.3 V (vs. Li^+^/Li). Our intensive theoretical/experiment sets based on the choice VST cathode architectonics modulated LIB models indicated that VST-(i)-type architectonic with predominant cave wavy canyons and long-range space domains of crater bays, coves, rims, and ridges as 3D accommodating space vacancy of electron/charge cloud configuration may offer a forest of electron mobility, short-range electron transport, and high-density ion diffusion in proximal massive craters, bays, and rims with dominated-rich surface faces and enriched upper-zone sites. VST-(i)@C cathode architectonics having vertical/central axes connected to four lateral/longitudinal exposure wings may also offer vast and broad surface access of Li^+^ ion loads in the axial, lateral, and horizontal directions for the movement of ions/electrons during the charge/discharge process. The VST-(i)@C hierarchical structure is a potential choice anode/cathode architectonics for high-performance full-scale LIB built-up models because it shows an excellent performance as a half-cell LIB with discharge capacities of 167.9 and 125 mAh g^−1^ at rates of 0.1 and 20 C, respectively, among all VST-(ii)-to-VST(Vii) architectonics [4]. The integration of superior choice anode/cathode architectonics in a full-LIB-scale built-up set (FRTO@C anode//VST-(i)@C cathode) configuration achieves a high long-term cycling performance (stability) with an excellent discharge capacity retention (~ 94.2%), an average Coulombic efficiency of 99.85% for 2000 cycles at a rate of 1 C, and a voltage range of 0.8–3.5 V at room temperature (Scheme 1). The proposed DFT surface analysis and building blocks for a full-scale battery system indicate the potential effect of the choice anode/cathode architectonics on high-performance full-scale LIB built-up models. The FRTO@C anode//VST-(i)@C cathode has an outstanding energy density of 127 Wh kg^−1^ that may satisfy the requirements for energy storage devices and meet the commercial requirements for EV applications.

## Experimental

### Controlled Synthesis of VST Cathodes

**Fig. 1:**
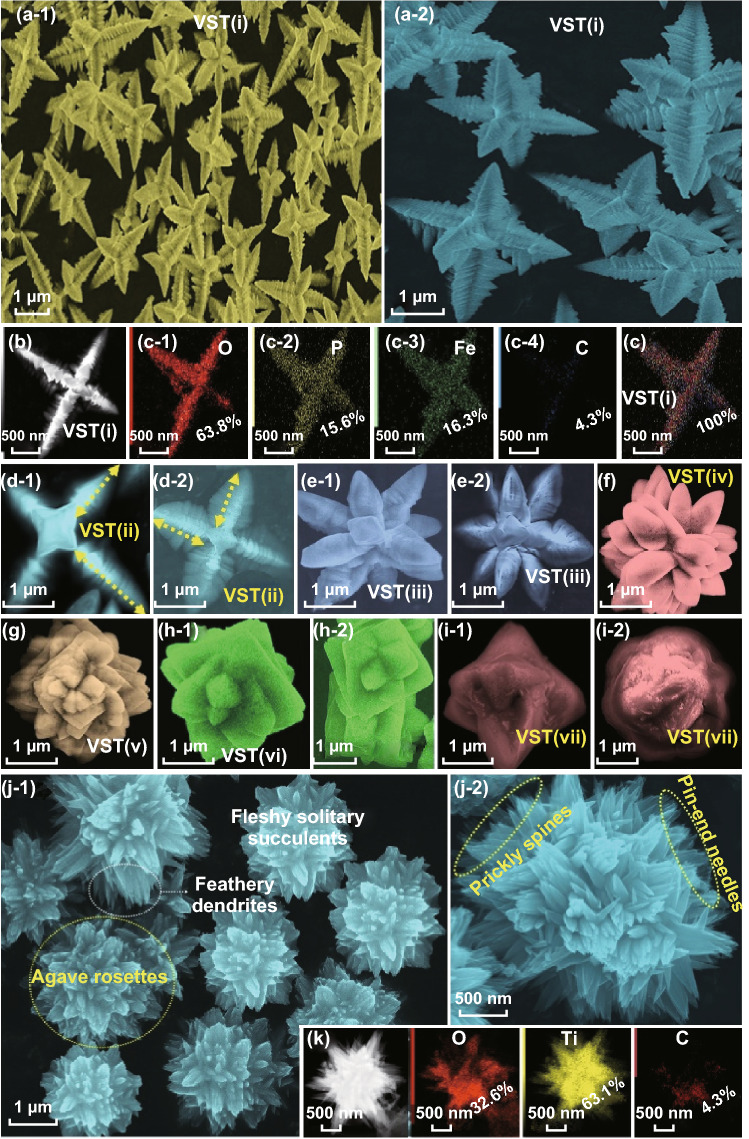
Low and high-resolution FE-SEM images, STEM-EDS pattern, and elemental mapping of heterogeneous composite superstructures of LiFePO_4_@C (cathode, **a**–**i**) and TiO_2_@C (anode, **j**–**k**) that are designated in vertical-star-tower (VST) building blocks and in closely packed flower agave rosettes (FRTO). **a**, **b** FE-SEM and STEM-EDS images of VST-(i)@C and (**d1**-**2**) VST-(ii)@C superpower hierarchy surfaces. **e**–**i** High-resolution FE-SEM of the (E)VST-(iii)@C, (F)VST-(iv)@C, (G)VST-(v)@C, (H)VST-(vi)@C, and (**i**-**1**, **i**-**2**) VST-(vii)@C power hierarchies with complex and stacked/condensed super structures blocks featured. **j**-**1** and **j**-**2** FE-SEM images, **k** Top view, bright field STEM image, and EDS elemental mapping of (FRTO@C) anode morphology that was designated in the flower agave rosettes hierarchy with closely packed solitary succulents, feathery prickly spines, and dendritic fleshy needle-ended branches

To control the VST anisotropic architecture complexity, a mixture of a VST cathode, labeled VST-(i), was prepared as follows. First, an aqueous solution mixture of phosphoric acid and 10 mL iron (III) nitrate nonahydrate (Fe(NO_3_)_3_·9H_2_O) was stirred for 1 h. Second, a 12.6 mL ethanol (Et):0 mL ethylene glycol (EG) mixture ratio (100%:0%) was added drop-wise (0.5 mL min^−1^) to the aqueous solution mixture under continuous stirring for 1 h. Third, a 10 mL lithium acetate dihydrate (CH_3_COOLi·2H_2_O) dissolved in Milli-Q-water was stirred for 1 h at 30 °C and added drop-wise to multi-component mixtures at the same rate (0.5 mL min^−1^). The total Li/Fe/P molar ratio in the final mixture was 3:1:1. This final mixture was vigorously stirred for 6 h to obtain VST-(i). The other VST mixtures (ii–vii, where i to vii denoted the range of anisotropic degree in architecture complexity) were synthesized following the same protocol but with the controlled addition of different volumes of Et/EG mixture ratios. The additive volumes of Et/EG (mL:mL) were 10.5:2.1, 8.4:4.2, 6.3:6.3, 4.2:8.4, 2.1:10.5, and 0:12.6, which correspond to Et/EG mixtures ratios of 83.33%:16.67%, 66.67%:33.33%, 50%:50%, 33.33%:66.67%, 16.67%:83.33%, and 0:100% for fabricating VST-(ii), VST-(iii), VST-(iv), VST-(v), VST-(vi), and VST-(vii), respectively (Fig. 1). The final mixtures of VST-(i), VST-(ii), VST-(iii), VST-(iv), VST-(v), VST-(vi), and VST-(vii) were transferred into 100 mL Teflon-lined stainless steel autoclaves and kept at 170 °C for 12 h before being cooled to room temperature. The resulting solid products were centrifuged, repeatedly washed with Milli-Q-water and absolute ethanol, and dried overnight at 60 °C under a vacuum. The VST was calcined in a muffle furnace under Ar at 450 °C for 6 h. The final VST powders were labeled VST-(i), VST-(ii), VST-(iii), VST-(iv), VST-(v), VST-(vi), and VST-(vii) architectonics and applied to cathode electrode fabrication; please see Supporting Information S1–S14.

### Synthesis of the TiO_2_ Flower Agave Rosettes Hierarchy

A150 mg titanoxysulfat-TiO(SO_4_)·xH_2_O was dissolved in 2 M hydrochloric acid (2 mol L^−1^) in 40 mL Milli-Q-water and stirred for 1 h. A 6 mL hydrogen peroxide (H_2_O_2_) solution was added drop-wise at rate 0.5 mL min^−1^ under vigorous magnetic stirring for 2 h. The final FRTO mixtures were transferred into a 100 mL of Teflon-lined stainless steel autoclaves and maintained at 170 °C for 12 h and cooled to room temperature. The resulting FRTO solid product was calcined in a muffle furnace under Ar at 450 °C for 6 h. In general, the drop-wise addition of H_2_O_2_ to TiO(SO_4_) contributes to the formation of a flower sphere-like vase with solitary succulents and feathery prickly spines. This time-dependent treatment leads to the formulation of agave rosettes with fleshy needle-ended branches (Fig. 1j, k). The FRTO materials were used for fabrication of the anode electrode. The FRTO@C electrode was also used for fabrication of the designated half-cell anode (2032 coin-type half-cell tests); please see Supporting Information S1, S14, and S15.

### Fabrication of the VST@C Cathode and FRTO@C Anode

The microwave-assisted approach was applied to formulate the superstructural hierarchy vertical star tower VST//flower agave rosettes FRTO nano-architecture. In accordance with typical synthesis, glucose (5 wt%) was added to as-prepared VST and FRTO samples. The glucose-dressing VST and FRTO samples were ground, added to Milli-Q-water/ethanol, and then ultrasonicated for 15 min. The mixtures were transferred to autoclaves and stirred for 30 min under microwave irradiation at 80 °C. The VST@C and FRTO@C precipitates were collected under centrifugation, washed by Milli-Q water and then ethanol, and then dried overnight at 55 °C. The resulting samples were calcined in an Ar atmosphere at 350 °C for 0.5 h and then maintained at 600 °C for 2 h with a heating rate 5 °C min^−1^. The final products were labeled VST-(i)@C, VST-(ii)@C, VST-(iii)@C, VST-(iv)@C, VST-(v)@C, VST-(vi)@C and VST-(vii)@C, and FRTO@C. The carbon coating of the VST (i–vii)@C cathode and FRTO@C anode structures reduces the nanoparticle size of C-dot bump maps and realizes a continuous distribution of the C-dot bump shell in a few nanometers (≤ 5 nm) (Fig. 1b, c, k). Various electrochemical experiments have been carried out on VST-(i) and VST-(i)@C half-cell cathodes to confirm the effect of well-ordered decoration and sustainable coating of C-shell dressers along super-hierarchal shaped VST cathodes (see Supporting Information S15). Indeed, the C-shell dressers along the superhierarchically shaped VST cathode and FRTO anode improved the kinetics of electron/Li^+^ ion transportation during the lithiation/delithiation process, as shown in Supporting Information S16 and S17.

### Choice Anode/Cathode Architectonics in Power Hierarchy LIB Built-up Sets

The power hierarchy VST@C//FRTO@C built-up electrode sets of the cathode//anode building blocks designated in CR2032 coin cells for both half-and full-LIB powers were controlled under specific protocols as reported in Supporting Information S1. Evidence of the retention of the topography and morphology of anode/cathode architectonics during the modulation of half-/and full-scale LIB designs was acquired by carrying out sets of theoretical and experimental analyses. Density functional theory (DFT) and electrostatic potential and electron maps (ESP–EM) of power hierarchy VST-(i–vii)@C cathodes and FRTO@C anodes were also studied according to DMol3 of the BIOVIA Dassault systems (see Supporting Information S2).

To explore the effect of a uniformly LFPO@C cathode that has morphological vertical-star-tower VST@C structures and is designated with flexible, multiple building blocks and units at high-end tower roofs on the LIB performance, we studied the non-uniform LFPO@C cathode in the electrochemical reaction of half-cell cathodes (see Supporting Information S16). The electrochemical performance, high capacity at high rate capability, and long cycle life of VST-(i)@C and non-uniform LFPO@C cathodes were studied in Supporting Information S16.

We intensively provided full studies to determine the optimized mass loading of full-cell (balancing) anode/cathode architectonics, named as *N*/*P* ratio (where *N* is a negative FRTO@C electrode capacity and *P* is a positive VST-(i)@C electrode capacity in mAh,) in the fabrication of powerful hierarchy LIB built-up sets; please see Supporting Information S1, S19, and S20. In this study, our proposed 3D super-scale stacked TiO_2_-based anode//LiFePO_4-_based cathode pouch LIB model was fabricated under an optimized mass loading of 13 and 6.9 mg cm^−2^ for the cathode and anode active materials, respectively. Moreover, the areal discharge capacities were 1.13 and 1.19 Ah cm^−2^ for the cathode and anode electrodes, respectively. These values were based on the fact that the discharge specific capacity (in Ah) should be approximately similar for both negative anode and positive cathode electrodes, as evidenced from the (*N*:*P*)_Cap_ capacity ratio of 1.05:1. In order to obtain the optimal trade-off full-scale LIBs in terms of better safety, the oversizing of negative electrode capacity is preferably needed to maximize the (*N*:*P*)_Cap_ capacity ratio so that it is close to a 1:1 ratio. Meanwhile, the optimum specific energy for our stacked pouch LIB model can be obtained at equal capacities along negative and positive electrodes, leading to an (*N*:*P*)_Cap_ capacity ratio of 1:1; please see Supporting Information S18–S20.

## Results and Discussion

### Superior Choice Anode/Cathode Architectonics in Full-LIB-Scale Built-up Sets

The powerful built-in LIB full cell design configuration, including its electron/Li^+^-ion diffusion capability, interplaying electron accommodation dominances, and electrochemical reactivity, was based on the superior choice anode/cathode architectonics in full-LIB-scale built-up sets. As a mode of choice anode/cathode architectonics in LIB design, we build novel 3D modulate LIB superstructural models based on amounts of uniformly cathodic LFPO@C vertical star towers with flexible, multiple building blocks and units at high-end tower roofs designated as VST-(i), VST-(ii), VST-(iii), VST-(iv), VST-(v), VST-(vi), and VST-(vii) architectonics. In addition, we used TiO_2_@C flower agave rosettes (FRTO@C anode) nano-architecture building to guarantee their outstanding electrochemical performance in half- and full-scale LIB designs (Scheme 1). To further understand the growth mechanism of anode/cathode architectonic crystals with various 3D-morphological architectures, typical factors, including the nucleation-directing agent and reagents insertion rate, are investigated (see Supporting Information S3). In general, chemically controlled composition domains can increase the number of VST block layers and cover the outer entrances, vacancies, grooves, and wings of the entire building block, thereby diminishing all possible free-movement electron, gateways, and diffusion pathways (Figs. S2–S7).

**Fig. 2:**
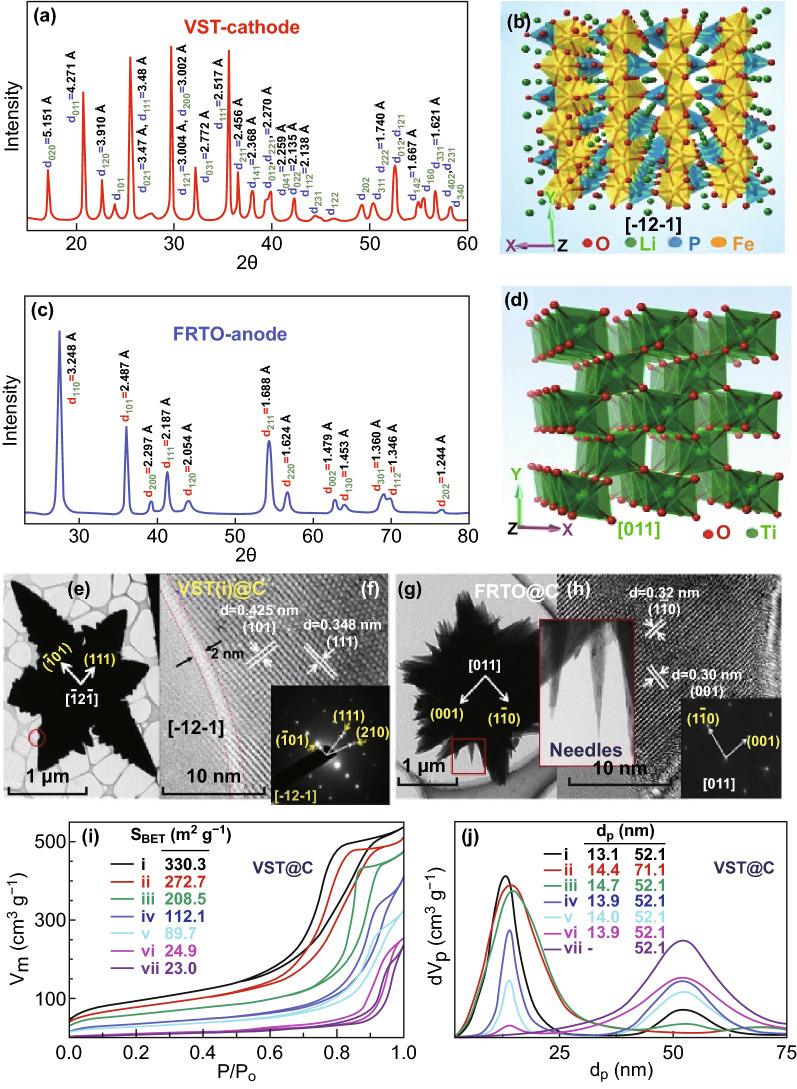
**a**, **c** XRD patterns of the VST-(i) cathode and FRTO anode hierarchy. **b**, **d** Crystal orientation VST cathode with orthorhombic-[− 12 − 1]-LFPO and the FRTO anode with rutile [011]-TiO_2_ structures. **e**, **g** Top view, low magnification HR-TEM micrographs of the VST-(i)@C cathode and FRTO@C anode. **f**, **h** High magnification HR-TEM micrographs of the VST-(i)@C cathode and FRTO@C anode oriented along [− 12 − 1] and [011] planes, insets of **f** and **h** are selected area electron diffraction patterns. **i** N_2_ adsorption–desorption isotherms of the structured materials featured a meso-cage accommodating space vacancy, (insert) surface parameters of surface areas in m^2^ g^−1^, and **j** pore size distribution curves, including pore diameters (dp/nm) for [VST-(i)@C, VST-(ii)@C, VST-(iii)@C, VST-(iv)@C, VST-(v)@C, VST-(vi)@C, and VST-(vii)]@C cathode structures

**Scheme 2:**
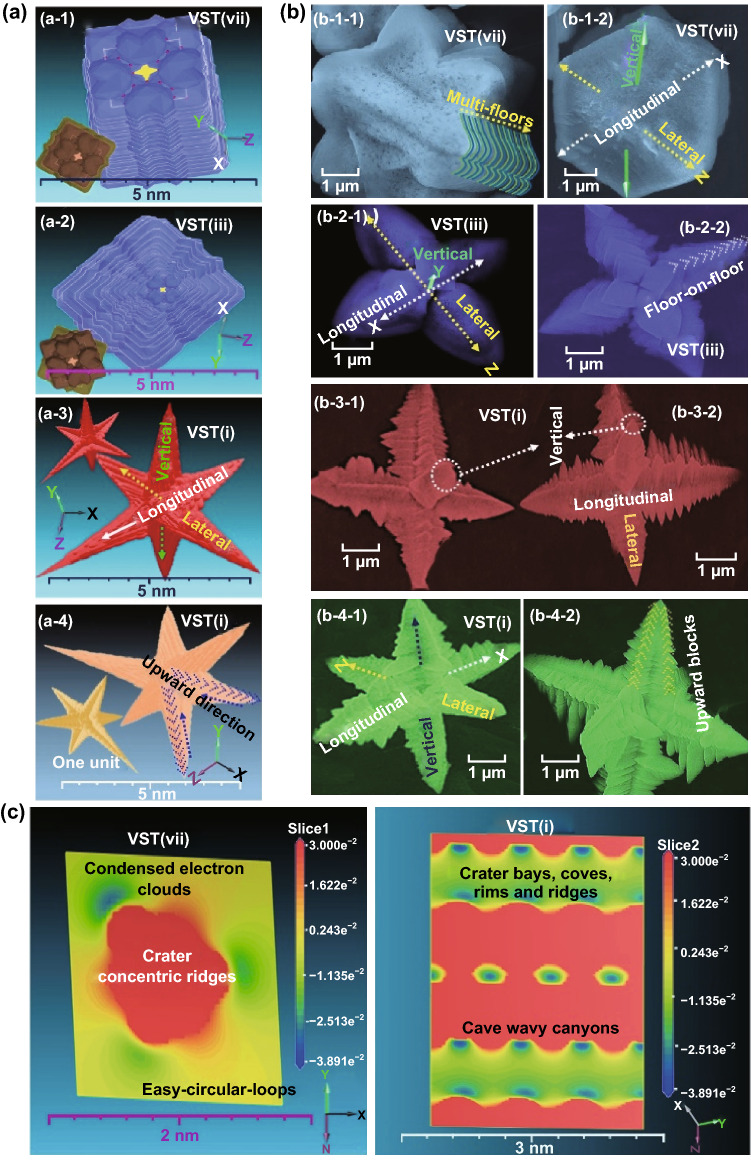
**a**–**c** 3D super-scalable model, FE-SEM micrographs, and electrostatic potential energy maps (ESP–EM) of the superstructural VST hierarchy with different building blocks. **a**-**1**-to-**a**-**4** Top-view, 3D modeling projection of modulate superstructural, vertical star tower building and hierarchal configuration VST-vii and VST-iii of a block/stacked tower with mesh mosaic, hexagonal prism, and cuboid structures (**a**-**1**, VST-vii), and with pyramidal apex surfaces (**a**-**2**, VST-iii); however, the 3D modulate super-star-structure VST-i (**a**-**3** and **a**-**4**) represented the top and side views of VST-i with open-end vestibule corridors, triangular pyramid grooves, multiple blocks in the upward direction, V-cave vacancies, and overall multi-orientation components oriented from the vertical spindle/central axis to 3D directions. (**c**-left, **c**-right) The electrostatic potential and electron maps (ESP–EM) and (+ve or –ve range) isosurface potential density and charge distribution of modulate crystal structures projected along the top- and central-zone edge and parallel to the c-axis orientation of superstructured surfaces of blocked/condensed VST-vii axes, and the open-axes of vestibule corridors of VST-i

The stability of the entire VST and FRTO super hierarchy backbone without an atomic-scale dislocation in the single-crystal orthorhombic olivine-type structure of the LFPO with the space group of Pnma (Figs. 2a, b, S10) and the tetragonal rutile TiO_2_ structure (Fig. 2c, d) was evident. The formation of the super-scale hierarchical VST@C cathode and FRTO@C anode surfaces indicates the following criteria: (1) a highly stable backbone surface coverage for assessing the frontal edge/apex/central surfaces (Fig. 2e, g) as revealed by the thermogravimetric analysis results, Fourier transform infrared spectroscopy, Raman spectra, and X-ray photoelectron spectroscopy (Figs. S9–S12) [25, 35, 36]; (2) stable surface active sites with low binding energy along the 3D affordable outer-, upper-, and middle-zone crystal cathode/anode surfaces (Fig. S14); (3) the formation of cross-linking network meshes of VST@C and FRTO@C via the intercalation C=C, C=N, and C=O bonds as revealed by the Raman analysis and the formation of actively oriented C–D- and C–G-type peaks; (4) the enhancements in the electronic conductivity and electrostatic electron/charge mobility of the VST@C cathode and FRTO@C anode surfaces; and (5) the formation of crater electron clouds and charge movements on the anisotropic potential heterogeneous surface, which leads to the configuration of a surface potential with wavy canyon-shaped loops (Scheme 2). Enhancing the surface potential of heterogeneous multi-component anode/cathode super-scale models will lead to the predominance of high-mobility electron/ion flows in full-scale LIBs.

The close resolution topography of transmission electron microscopy (TEM) patterns along the VST@C and FRTO@C super-scale hierarchies reveal bowl-shaped ridges/alcoves along the lateral, vertical, and longitudinal convex-up planar needles and carved trenches in the flanks at the in–out-plane of the upper-zone surface of open-multi-directional orientations of VST wings, corridors, and groves, and FRTO agave rosettes with closely packed solitary succulents, feathery prickly spines, and dendritic fleshy needle-ended branches (Fig. 2e, g). The microscopic objects recorded along all directions of the entire VST@C and FRTO@C reveal the formation of craving host-caved nest architectures for a dynamic environment and can accommodate a seamless transition of vacancy channels and tunnels. The HR-TEM image of VST-(i)@C shows a clear pattern of crystal planes with d-spacing values of 0.425 and 0.348 nm, which correspond to the (− 101) and (111) planes of the orthorhombic LFPO domain [− 12 − 1] plane, respectively (Fig. 2f and inset). In addition, the FRTO@C displays a clear pattern of crystal planes with d-spacing values of 0.30 and 0.32 nm, which correspond to the (001) and (− 110) planes of the predominate rutile [011]-TiO_2_ plane (Fig. 2h and inset). The 3D microscopic and crystal surface analyses (Figs. 1, 2, S11–S14) present some evidence on the actively energetic surface coverage of the super-scalable VST@C and FRTO@C cathode/anode, thereby making this material a promising candidate that offers a high electron/ion flow mobility, discharge capacitance, and enhancement surface potential during the charge/discharge process.

Figure 2i, j presents the N_2_ adsorption–desorption isotherms of mesoporous structures incorporated with an interiorly dense kernel cavity with abundant cave spaces along the bays, rims, and ridges to achieve a facile diffusion mobility and suitable accommodation of Li^+^ ions during the charge/discharge process [37–40]. The large surface area of the super-scalable VST@C and FRTO@C can lead to the creation of multi-diffusible cathode/anode electrode packages. The vertically geometric VST@C cathodes oriented with the confinement open-end vestibule corridors of VST-(i) and (ii) and with a dense stacking of the layer-to-layer building block VST-(iii, iv, v, vi, and vii) morphologies affect the surface area-to-volume ratio and the overall surface coverage of the cathode electrode in terms of its (1) accessible electron/Li^+^ ion accommodation, (2) high loads/diffusion/storage, and (3) thermodynamically stable surface energy.

### Theoretical DFT Analysis

To explore the effective fabrication of (1) the 3D super-scalable model structure and its building along the axial orientation, (2) the surface potential configuration and density, and (3) the charge distribution of modulate crystal structures on the potential full-scale LIB design, we performed intensive surface-to-surface electronic and charge map analyses of the cortical cathode electrode by using electrostatic potential and electron maps (ESP–EM). We also analyzed the (+ve or –ve range) isosurface potential density and 3D models of crystal building projections by performing DFT calculations of representative VST@C cathodes [40–42], including VST-block/stacked [i.e., VST-(iii) or VST–(vii)] and VST-open-end vestibule corridors [i.e., VST-(i) or VST-(ii)], as shown in FE-SEM images (Scheme 2b1–b4). A set of 3D modeling projections of modulate floor-on-floor building blocks was built-in 3D axes overlying sides and faces in dense upward layers that shaped like vertical star tower VST-(Vii)@C, VST-(iii)@C, and VST-(i)@C with lateral, and longitudinal orientation wings concentrically connected at the center feed points of the main vertical axis (Scheme 2a-1 to a-4, b-1 to b-4) to create closely packed three-directional-integral dipole multiples for a widespread “radial, lateral, and longitudinal” diffusion of ions/electrons to minimize surface resistance and to reduce the diffusion distance along the electrode pattern. A 3D modeling hierarchical configuration of block/stacked VST-(Vii)@C and VST-(iii)@C has a mesh mosaic, hexagonal prism, and cuboid structure (Scheme 2a-1) with pyramidal (Scheme 2a-2) apex, edge, and vertex-ended surfaces. Furthermore, the VST-(i)@C open-end vestibule corridors have a single upper-top-capped pyramidal prism unit that heads toward the end-zone incidents of each axial, lateral, and longitudinal wing scale. These capping prism-based shapes have a clear apex, edge, and vertex-ended surfaces to ensure excellent and maximum directional diffusion throughout all dimensions and loops. The top-view FE-SEM and 3D topographic VST-(i)@C images along the upward direction and along all sides of the lateral, vertical, and longitudinal axes have bowl-V-shaped ridges/alcoves, extensive convex-up planar needles arranged in a tip-to-tip configuration, and carved trenches in the flanks at the in–out-plane of the upper zone surface of open-multi-directional tower wings (Scheme 2a, b-3, b-4), which lead to the predominance of vast transport and electron/ion mobility flows along all possible surface potentials.

Scheme 2c-1, c-2 presents the ESP-EM of the superstructural hierarchy as well as the (+ve or –ve range) surface potential density and charge distribution of the modulate crystal structures that are projected along the top- and central-zone edges and parallel to the *c*-axis orientation of the superstructured surfaces of block/stacked VST-(Vii)@C (Scheme 2c-1) and VST-(i)@C open-end vestibule corridors (Scheme 2c-2). The ESP-EM topography shows that the upper-top, stacked VST-(vii)@C has condensed electronic phoenix clouds with an actively energetic surface coverage (Scheme 2c-1) and non-ward-trending, non-dimensional electron mobility pathways. In turn, the surface coverage of VST-(i)@C open-wings and multi-directions shows an in-to-out electron dominance distribution in crater shapes, such as bays, coves, and rims. The electron cloud arrangement scales along the upper, middle, and lower multi-zone surface patterns have cave wavy canyons for assessing the excellent specific capacities, facile charge–discharge rates, high energy density, and long-term stability of the frontal edge/apex/central surfaces for their (Scheme 2c-2). The canyon, distal wavy cage-like clouds of the planar-bedded electron surface configuration showed a forest of electron mobility of proximal massive cages that dominated-rich surface faces and enriched upper-zone sites. The potential configuration and charge distribution with a calculated isosurface value is 0.0089, with a range of − 0.0389 to 0.030 for the ESP. The surface charge tendency and canyon cage-like clouds result in giant focal diffusion of electrons/ions dominance mobility along entirely peripheral top electrode surfaces.

With the manufacturing control of harmonious architectural LIB super-star-built towers, the key components of open and dense scalable mosaic towers, the 3D accommodating space vacancy of the electron/charge cloud configuration, and the multi-directional orientation components are investigated during the control design of power hierarchy VST-(i)@C//FTOR@C full-cell LIB built-up sets. We also evaluate the effectiveness of the scalable architectures of VST-(i), VST-(ii), VST-(iii), VST-(iv), VST-(v), VST-(vi), and VST-(vii) as cathodes that improve the electrochemical performances of the half-cell LIB model. To design a full-scale anode/cathode LIB modulation, we also evaluate the electrochemical performance of the FTOR@C-anode as a half-cell LIB (Fig. S13). Our intensive theoretical/experiment sets based on the choice anode/cathode architectonics modulated LIB models lead to an increase in energy density, ensure long-term stability, and enhance the surface potential relaxation of LIBs to ensure outstanding rate discharge capacities.

### Half-Cell Design Based on Power Hierarchy VST-(i–vii)@C Cathodes

Cyclic voltammetry (CV) measurements were performed to compare the electrochemical performance of the VST-(i)@C composite with those of other power cathode VST-(ii–vii)@C hierarchies in the half-cell LIB model.

**Fig. 3:**
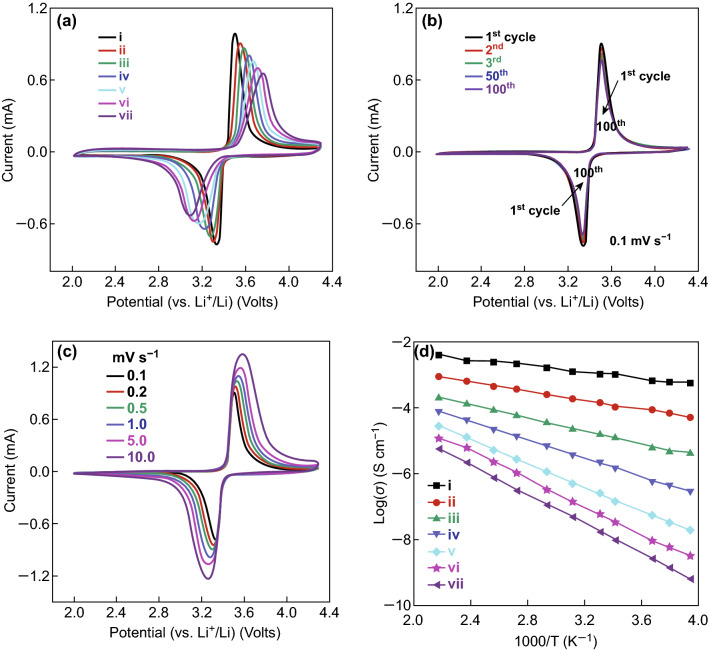
**a**-**c** Cyclic voltammograms (CVs) associated with a comprehensive investigation of the electrochemical performance of the power cathode VST-(i–vii)@C hierarchy designated in the half-cell LIB model (2032 coin-type half-cell tests with a Li counter electrode). **a** CV curves of different VST-(i)@C, VST-(ii)@C, VST-(iii)@C, VST-(iv)@C, VST-(v)@C, VST-(vi)@C, and VST-(vii)@C cathodes. **b** CV curves of the VST-(i)@C cathode at different cycle numbers that varied from 1 to 100 cycles at 0.1 mV s^−1^ in a half-cell LIB and at different sweep rates (0.1, 0.2, 0.5, 1, 2, and 5 mV s^−1^). **c** All electrochemical measurements for the half-cell VST-(i)@C cathodes were operated within a voltage range of 2.0–4.3 V at room temperature. **d** Temperature dependence comparison of electrical conductivity for different structures of power cathode VST-(i–vii)@C hierarchy

Figure 3a presents the cyclic voltammogram curves for a mount of a VST@C multi-component hierarchy with open-ended directions of VST-(i) and VST-(ii) and the blocked/stacked VST-(iii) to VST-(vii) blocks within the potential region of 2–4.3 V versus Li/Li^+^ at scan rate of 0.1 mV s^−1^. The oxidation/reduction peaks for VST-(i), VST-(ii), VST-(iii), VST-(iv), VST-(v), VST-(vi), and VST-(vii) are located at 3.34/3.5, 3.31/3.55, 3.28/3.59, 3.23/3.63, 3.18/3.67, 3.12/3.71, and 3.07/3.76 V, respectively. The cyclic voltammograms of VST-(i)@C for the 1st, 2nd, 3rd, 50th, and 100th cycles at 1 mV s^−1^ within the potential region from 2.0 to 4.3 V are presented in Fig. 3b. The VST-(i)@C cathode shows symmetric and spiculate Fe^2+/^Fe^3+^ peak profiles at 3.34/3.5 V, thereby highlighting the effects of 3D super-star-tower VST-(i) scales on the facile reversible electrochemical reaction during the Li^+^ ion insertion (i.e., cathodic, reduction, or lithiation) and extraction (i.e., anodic, oxidation, or delithiation) processes [43]. The overlapping of CV curves during the charge/discharge process is attributed to the excellent cycling performance of VST-(i)@C, which exhibits a high reversible capacity even at 100 cycles. Figure 3c presents the typical 1st cyclic voltammogram profiles of the VST-(i)@C half-cell cycled at scan rates of 0.1, 0.2, 0.5, 1, 2, 5, and 10 C between 2.0 and 4.3 V versus Li/Li^+^ at a scan rate of 0.1 mV s^−1^. The temperature dependence of the electrical conductivity of different VST@C cathode structures is compared in Fig. 3d. All VST (i–vii) samples with different morphologies exhibit good conductivity over a temperature of 298 K. Among all super-star-tower cathodes in the half-cell-LIB, the VST-(i)@C hierarchy shows the best conductivity across all tested temperatures (− 250 to 455 K). The finding reveals the hotkeys of the three-directional ordering of VTS-(i)@C to offer diffusion of Li^+^ ions/electrons along the concentrically connected feed points at the center axis. Furthermore, VST-(i)@C cathode design featured non-resisting spread electrons/Li^+^ ion loads along the integral dipole multiples and its rich spatial distribution complexity extended in a widespread directivity along radial, lateral, and longitudinal axes. These architectural features minimize the surface resistance and decrease the diffusion distance along the VTS-(i)@C electrode pattern compared with other VST@C profiles.

**Fig. 4:**
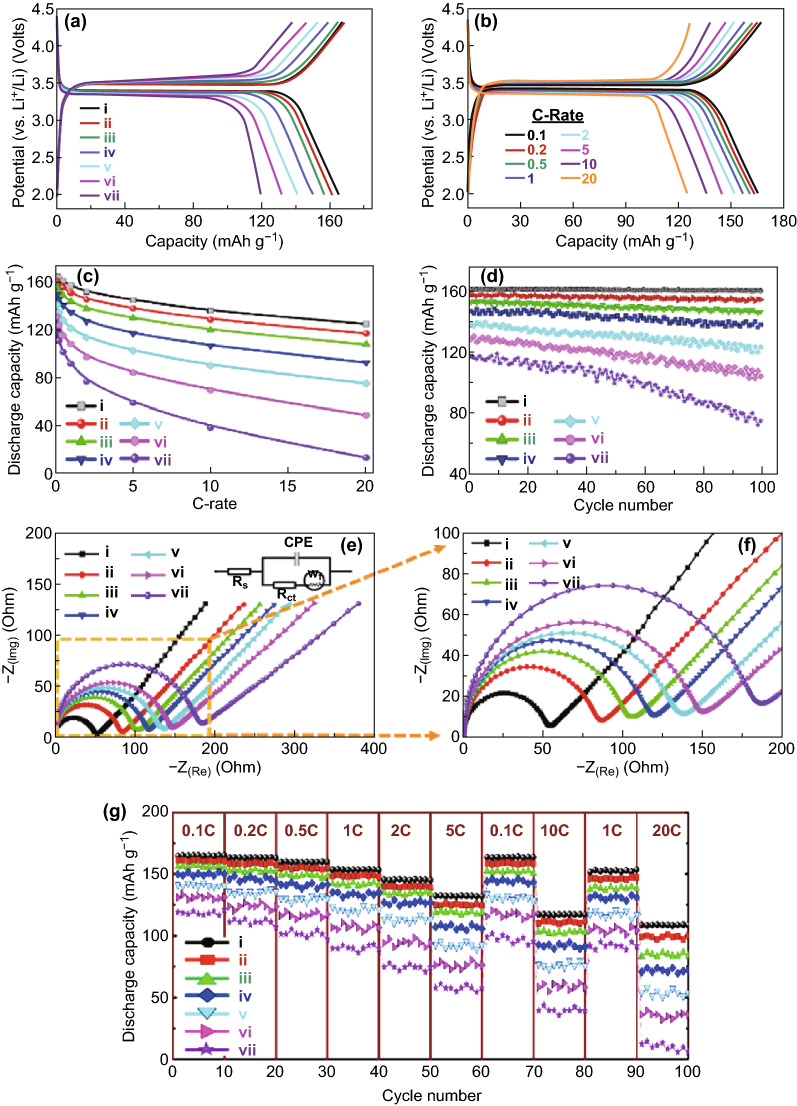
**a** Charge–discharge voltage profiles of first cycle current rate 0.1 C half-cell of the power cathode VST-(i–vii)@C hierarchy designated in the half-cell LIB model (2032 coin-type half-cell tests with a Li counter electrode). **b** The VST-(i)@C hierarchy designated in the half-cell LIB model at the first cycle comparison between half-cells at different current rates varying from 0.1 to 20 C. **c** First discharge capacity of all power cathode VST-(i–vii)@C hierarchies designated in the half-cell LIB model at a current rate of 0.1 C. **d** Cycling performance (stability) for all power cathode VST-(i–vii)@C hierarchies designated in the half-cell LIB model at a rate of 0.1 C for 100 cycles. **e** The electrochemical impedance spectroscopy (EIS) result of the power cathode VST-(i–vii)@C hierarchy designated in half-cell LIB models; the inset of **c** is the equivalent circuit agreement with the impedance results of the EIS. **f** enlargement of the semicircle part of **e**. **g** Rate capability performance over a range of 2.0–4.3 V among all tested LFPO@C cathodes of different structures at various current rates from 0.1 to 20 C. All electrochemical measurements were operated within a voltage range of 2.0–4.3 V at room temperature

Figure 4a shows the cycling performances of the VST-(i)@C, VST-(ii)@C, VST-(iii)@C, VST-(iv)@C, VST-(v)@C, VST-(vi)@C, and VST-(vii)@C cathodes within the potential region of 2 to 4.3 V versus Li/Li^+^ at a sweep rate of 0.1 mV s^−1^. The VST@C half-cells were charged to 4.3 V at 0.1 C, maintained at 4.3 V for 1 h, and then discharged to 2.0 V at 0.1 C. Among all cathodes, VST-(i)@C shows the highest storage capacity. At 0.1 C, VST-(i)@C shows a high discharge capacity of 167.9 mAh g^−1^, while VST-(ii)@C, VST-(iii)@C, VST-(iv)@C, VST-(v)@C, VST-(vi)@C, and VST-(vii)@C have high discharge capacities of 161, 156.2, 149.8, 141, 131.4, and 119.1 mAh g^−1^, respectively. Figure 4b presents the typical 1st cycle voltage profiles for VST-(i)@C cycled at scan rates of 0.1, 0.2, 0.5, 1, 2, 5, 10, and 20 C between 2.0 and 4.3 V. The specific discharge capacities in mAh g^−1^ versus the current C rates from 0.1 to 20 C for the half-cell VST-(i)@C, VST-(ii)@C, VST-(iii)@C, VST-(iv)@C, VST-(v)@C, VST-(vi)@C, and VST-(vii)@C cathodes are shown in Fig. 4c. The VST-(i)@C cathode exhibits an excellent discharge capacity at C rates ranging from 0.1 to 20 C compared with the other VST@C half-cells. The superior long-term cycling performance and stability of the current VST@C cathodes are presented in Fig. 4d. VST-(i)@C retains 99.5% of its 1st cycle capacity after 100 cycles at 0.1 C. Meanwhile, VST-(ii)@C, VST-(iii)@C, VST-(iv)@C, VST-(v)@C, VST-(vi)@C, and VST-(vii)@C retain 98.0%, 95.9%, 94.0%, 89.1%, 80.9%, and 63.7% of their initial capacities after 100 cycles at 0.1 C, respectively. The VST-(i)@C half-cell cathode does not demonstrate any capacity fading over 100 cycles at a rate of 0.1 C, revealing the hotkeys of the open-star tower VST-(i)@C hierarchy in high electrochemical reversibility during the lithiation/delithiation process.

The wing directions in the lateral, vertical (axial), and longitudinal axes have bowl-shaped ridges/alcoves, extensive convex-up planar needles arranged in a tip-to-tip configuration, and carved trenches in the flanks at the in–out-plane of the upper zone surface of open-multi-directional tower wings, all of which result in the predominance of high-mobility electron/ion flows and the enhancement of surface potential. The single capping of the rectangular-top-head pyramid grooves have clear apex, edge, and vertex-ended surfaces to ensure an excellent and maximum directional diffusion throughout all dimensions and loops compared with the stacked-dense blocks of VST-(iii) to VST(vii) buildings (Scheme 2).

To explore the effect of (1) well-ordered decoration and sustainable coating of C-shell dressers along super hierarchically shaped VST cathodes and (2) a uniformly ordered LFPO@C cathode that has a morphologically vertical star tower VST@C structures on the improvement of the kinetics of electron/Li^+^ ion transportation during lithiation/delithiation, we conducted various electrochemical experiments on (1) structurally non-controlled LiFePO_4_@C and (2) both hierarchically controlled VST(i) (i.e., non-coated carbon) and (3) VST(i–vii)@C (i.e., coated carbon) cathodes (Figs. 4e, f, S15, S16, and Table S2). For instance, Nyquist graphs show a semicircle at the high-frequency region and a slanted line at the low-frequency region (Figs. 4e, f, and S16). The electrochemical impedance spectroscopy (EIS) results were determined according to the equivalent circuit, as shown in the inset of Figs. 4e and S16. The equivalent circuit comprises an electrolyte resistance (Ohmic resistance *R*_s_, which refers to the intercept impedance on the real axis and corresponds to solution resistance), charge transfer resistance (*R*_ct_, which results from the electrochemical interaction at the electrode/electrolyte interface and particle/particle contact), constant phase element, and Warburg impedance of the Li^+^ ion diffusion into the electrode (*W*_f_; which is related to the low-frequency region of the straight line) [25, 44–47]. The semicircular arc at the highest frequency range is relative to the *R*_ct_ value, which is approximately equal to the numerical value of the diameter of the semicircle on the *Z* real axis. A constant phase element CPE was placed to represent the double layer capacitance and passivation film capacitance. In general, the LIB cell impedance is primarily attributed to both functions of (1) cathode impedance and (2) charge transfer resistance. The exchange current density of the LFPO electrode (*I*_0_) can be calculated (Table S2) according to Eq. 1 [25]:

1 $$I_{0} = \, RT/nFR_{\text{ct}}$$

where *R* represents the gas constant (8.314 J mol^−1^ K^−1^), *T* is the absolute temperature (298 K), *n* is the relevant number of electrons in the LFPO redox reaction (*n* = 1 for Fe^2+^/Fe^3+^ redox pair), and *F* is the Faraday constant (96,485 C mol^−1^).

The calculated values of *R*_s_, *R*_ct_, and *I*_0_ of the tested cathode samples agreed well with the measured values. Table S2 shows the parameters of the equivalent circuit for (1) structurally non-controlled LiFePO_4_@C and (2) both hierarchically controlled VST(i) (i.e., non-coated carbon) and (3) VST(i-vii)@C (i.e., coated carbon) cathodes. These *R*_s_, *R*_ct_, and *I*_0_ parameters were obtained from computer simulations using ZAHNER-Elektrik v. 3.0 software. The resistance of the electrolyte/electrode (*R*_s_) is very similar for all cathode samples because the addressing of carbon shells significantly enhances the conductivity of all electrodes. The *R*_ct_ of the VST(i)@C cathode showed the smallest semicircle diameter and a lower value (i.e., 54.5Ω) than those of other cathodes. The exchange current density (*I*_0_) of VST(i)@C composite is higher than those of the other cathodes. This finding indicates the key components of the building blocks of cathodes with vast active surfaces and superhierarchically shaped and uniformly ordered structures in powerful in-built half-cell LIB cathodes that offer excellent electron/ion transfer kinetics along cathode surfaces through the lithiation/delithiation process (see Supporting Information S16–S20).

The key factors of VST hierarchy blocks designated with overlying sides and faces in dense upward blocks, the open and widespread directivity of power hierarchy LIB built-up sets, the long-term stability of cycles, and the performance of VST@C must be investigated to meet the PHEV and EV application requirements (Fig. 4g). To evaluate the rate capability of VST@C-type cathodes, their cycling performance was tested at different rates (0.1, 0.2, 0.5, 1, 2, and 5 C then back to 0.1 and 10 C and then back to 1 and 20 C, with 10 cycles at each rate) at room temperature (Fig. 4g). The reversible discharge capacity of these cathodes significantly reduced to 108.6, 100.2, 83.7, 72.0, 54.0, 36.1, and 5.7 mAh g^−1^ during the repeated Li intercalation at a rate of 20 C over 100 cycles. In other words, the specific capacity usually decreases as the C rate increases for all tested cathodes. Among all cathodes in half-cell LIBs, the VST-(i)@C cathode exhibited a superior rate performance at a rate of 20 C after 100 cycles. The excellent electrochemical performance, high capacity at high rate capability, and long cycle life of this cathode may be ascribed to the hierarchy regular micromorphology and 3D superstructure of the VST-(i)@C composite with single, upper-top-capped pyramidal prism headings at the end-zone incidents of the multi-directional wing scales (Fig. 4g). The wings along the lateral, vertical (axial), and longitudinal axes exhibit bowl-shaped ridges/alcoves that result in the predominance of high-mobility electron/ion flows and enhancements in the surface potential. The capping prism-based shapes have clear apex, edge, and vertex-ended surfaces, which contribute significantly to the high volumetric energy density, excellent rate capability, and vast directional Li ion diffusion throughout all dimensions and loops of the VST-(i)@C cathode. By incorporating the onsite blocking/massing into the dense building component design [VST-(iii)-to VST-(vii)] along the dramatic tetra-partite wing, the tower assembly considerably (1) hindered in the dynamic profiles, (2) alleviated some kinetic movements, and (3) limited the supply of charge accommodating space and multiple-convergence vacancy rates, thereby leading to the low efficiency of electro-transition systems.

In summary, the remarkable advantages of VST-(i)@C, including its superb retention performance, excellent rate capability, and high cycling stability, can be attributed to (1) the rapid electron movement reaction and Li^+^ diffusion kinetics at interfaces during the lithiation/delithiation process; (2) the well-structured robustness, excellent electronic contact, and high electronic conductivity of the cathode as well as its ability to reduce the Li^+^ ions diffusion path, facilitate the transport of electrons, and improve electrical conductivity; (3) the 3D hierarchical super-scale VST-(i) building blocks with multi-diffusive meso/macro open sites; and (4) the open-multi-direction, scalable mosaic towers, which comprise a mass of curved, prismed room grooves of VST-(i) scales that are connected to corridor tunnels and evoke a crop of bowels, with each kernel cavity representing individual cave spaces for facilitating diffusion mobility and ensuring the accommodation of Li^+^ ions during the charge/discharge process. Given its outstanding electrochemical performance, VST-(i)@C is considered one of the most in-demand cathodes for meeting the high-power and high-energy requirements of LIBs and EV applications, as evidenced from Table S3 and Supporting Information S22.

### Half-Cell Design Based on the FRTO@C Anode Design

To show the 3D surface arrangement of the super-scalable FRTO@C anode design with a flower agave rosettes hierarchy and closely packed solitary succulents, feathery prickly spines, and dendritic fleshy needle-ended branches, we studied the typical galvanostatic cycling/voltage curves for the FRTO@C anode in half-cell configurations within the potential range of 1 to 3 V versus Li/Li^+^ at 1 C and 1, 5, 60, 100, and 200 cycles (Fig. S13a). First, each discharge profile demonstrates a rapid decrease in potential from the open circuit voltage (~ 3 V) to ca.1.7 V versus Li. Second, the discharge region represents a nearly distinct light slope plateau zone from 1.7 to 1.5 V, which is indicative of the lithiation process and corresponds to the two-phase reaction. Third, the region after the plateau zone displays a long gradual decrease in potential from 1.5 to the cutoff voltage 1.0 V versus Li, which may be explained by the large lithiation loading and accommodation in the multi-zone-surface vacancy of the FRTO@C anode (interfacial storage) [48]. The charging profiles also exhibit three definable characteristic stages. The first stage shows an increase in capacity from 1 to 1.96 V due to the monotonic delithiation process. The second stage is characterized by a continued Li^+^ extraction process through the regime from 1.96 to 2.25 V. The third stage represents a curved solid solution regime toward 3.00 V. The FRTO@C anode shows an irreversible capacity for the first five cycles, which is in agreement with the rutile-phase TiO_2_ anode (Table S4). This finding reveals the key component responsible for the growth of the FRTO@C solid-electrolyte interface (SEI) layer [49–51].

Figure S13b exhibits the good cycling performance and excellent Coulombic efficiency of the half-cell FRTO@C anode at a rate of 1 C within the potential window of 1 to 3 V at room temperature. Although the charge and discharge capacities of this anode have drastically decreased, its Coulombic efficiency has increased from the initial charge and discharge capacities of 231 and 329 mAh g^−1^, respectively. The initial Coulombic efficiency of 70.2% at 1 C has increased as the cycle increased to 40. After exceeding 40 cycles, the charge and discharge capacities remain almost the same but slightly decrease from 190 mAh g^−1^ at 40 cycles to ~ 172 mAh g^−1^ at 200 cycles. In turn, the Coulombic efficiency has increased from 98.5% at 20 cycles to 99.6% at 200 cycles.

To evaluate the rate capability performance of FRTO@C as an anode material in a range of 1.0 to 3.0 V, we examine its discharge cycling performance at 0.1, 0.2, 0.5, 1, 2, and 5 C, back to 0.1 and 10 C, and then back to 1 and 20 C (with 10 cycles at each rate) up to 100 cycles at room temperature (Fig. S13c). The specific capacity of FRTO@C decreases with an increasing current rate, while its capacity significantly decreases during the repeatable Li intercalation process to be 60 mAh g^−1^ at a rate of 20 C and after 100 cycles. After the first 10 cycles, the irreversible capacity loss is likely due to the electrolyte decomposition and formation of the stable solid-electrolyte interface (SEI) layer on the FRTO@C anode. In summary, the super-scalable FRTO@C anode design with the flower agave rosettes hierarchy and closely packed solitary succulents, feathery prickly spines, and dendritic fleshy needle-ended branches has vast active surfaces for the axial, lateral, and horizontal movements of Li^+^ ions/electrons during the charge/discharge process. To illustrate the effect of the hierarchically controlled FRTO@C anode structure on high half-cell anode performance, we conducted comparison studies between the FRTO@C half-scale LIB and the other reported TiO_2_ anodes, as shown in Table S4 (Supporting Information S23). The findings revealed the key attributes of hierarchically ordered FRTO@C architectonics affecting the performance of half-cell LIB anodes.

### Powerful Built-in Full-Cell Sets of VST-(i)@C//FRTO@C Integration

The high cycling performance of the designated half-cell-based FRTO (TiO_2_)@C anode and VST-(i) (LiFePO_4_)@C cathode super-hierarchical architectures is driven by our construction of a novel outstanding performance hybrid full-cell VST-(i)@C//FRTO@C LIB. We used powerful-in-built full-scale LIB-based VST-(i)@C//FRTO@C super-hierarchical cathode//anode building blocks designed in 2032 coin-type and stacked layers pouch full-cell LIB models. We also used Cu and Al foils as counter electrodes at supreme electrochemical performance conditions, as shown in Supporting Information S1. To help clarify the superior choices of (1) anode/cathode architectonics and (2) the full-cell LIB built-up configurations designated in cylindrical or stacked pouch models, calculation processes of specific energy density, capacity balancing with N/P ratios, and volumetric energy density of VST-(i)@C//FRTO@C full-scale LIB sets were intensively studied, as shown in Supporting Information S18–S20. Our finding supports the use of the full-cell 18,650-cylindrical design as a model for our proposed VST-(i)@C//FRTO@C material-based electrodes.

**Fig. 5:**
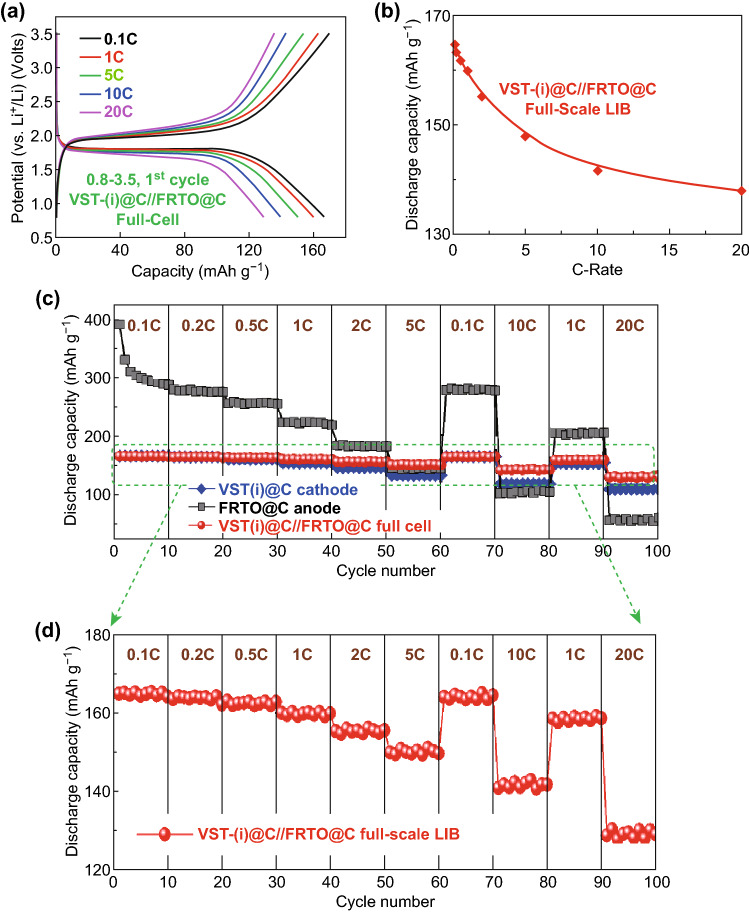
**a** Charge–discharge voltage capacity profiles of VST-(i)@C//FRTO@C full-scale LIBs at different current rates 0.1, 1, 5, 10, and 20 C. **b** Behavior of the specific discharge capacity versus current C rates varying from 0.1 to 20 C for a full-cell VST-(i)@C//FRTO@C LIB. **c** Performance and behavior of the rate capability for a powerful in-built super-hierarchal full-scale VST-(i)@C (cathode)//FRTO@C (anode) at various current rates from 0.1 to 20 C. **d** Enlargement of the selected part of **c** for rate capability performance results of the VST-(i)@C//FRTO@C full cell. All electrochemical measurements were operated within the voltage range of 0.8–3.5 V at room temperature

Figure 5a shows the 1st cycle charge–discharge voltage capacity profiles of VST-(i)@C//FRTO@C full-scale LIBs between 0.8 and 3.5 V at 0.1, 1, 5, 10, and 20 C at room temperature. The 1st cycle charge/discharge capacities were 169.3/166; 162/160; 153/149.8; 142/139.2; 135/128.5 mAh g^−1^ at 0.1, 1, 5, 10, and 20 C, respectively. The average initial Coulombic efficiency was approximately ~ 98.5% at different rates. Figure 5a also showed that the average working voltage of the battery, which is ~ 1.8 V, provides a specific energy density of 288 Wh kg^−1^ for the LiFePO_4_ (VST-(i)@C) cathode. Based on the mass fraction analysis of a commercial pouch LIB cell (Table S1), the mass fraction of a LFPO@C cathode material in a full-cell LIB is approximately 44%. Therefore, the practical value of a specific energy density for the VST-(i)@C//FRTO@C super-hierarchical building blocks full-scale LIB models was found to be 127 Wh kg^−1^. This specific energy density of the full-cell VST-(i)@C//FRTO@C LIB offers the driving range requirement for EVs. In a point-of-manufacture demand, the large-scale EVs pack design using a VST-(i)@C//FRTO@C full-scale system would be preferably designated with 18,650-cylindrical-shaped models, as shown by the intensive studies described in Supporting Information S18–S20.

To check the integration of the powerful built-in full-cell sets of VST-(i)@C//FRTO@C, we examined their specific discharge capacities across different scan rates (0.1, 0.2, 0.5, 1, 2, 5, 10, and 20 C) at a voltage range of 0.8 to 3.5 V (Fig. 5b). VST-(i)@C//FRTO@C exhibits an excellent discharge capacity over a wide range of current C rates, i.e., from 0.1 to 20 C. Figure 5b shows that the discharge capacities decrease with an increasing C rate, but reach 128.5 mAh g^−1^ at 20 C. A powerful in-built full-cell LIB exhibits a high specific energy density that overrides the requirement for long-term EVs with excellent power. Figure 5c, d rationalizes the outstanding rate capability of the hybrid VST-(i)@C//FRTO@C full-cell at 0.1, 0.2, 0.5, 1, 2, and 5 C, back to 0.1 and 10 C, and then back to 1 and 20 C (with 10 cycles at each rate) up to 100 cycles over a voltage range of 0.8 to 3.5 V at room temperature. The specific discharge capacity of the VST-(i)@C//FRTO@C full-cell LIB decreases with an increasing current rate. This finding indicates the outstanding rate capability of the hybrid VST-(i)@C//FRTO@C at different rates.

**Fig. 6:**
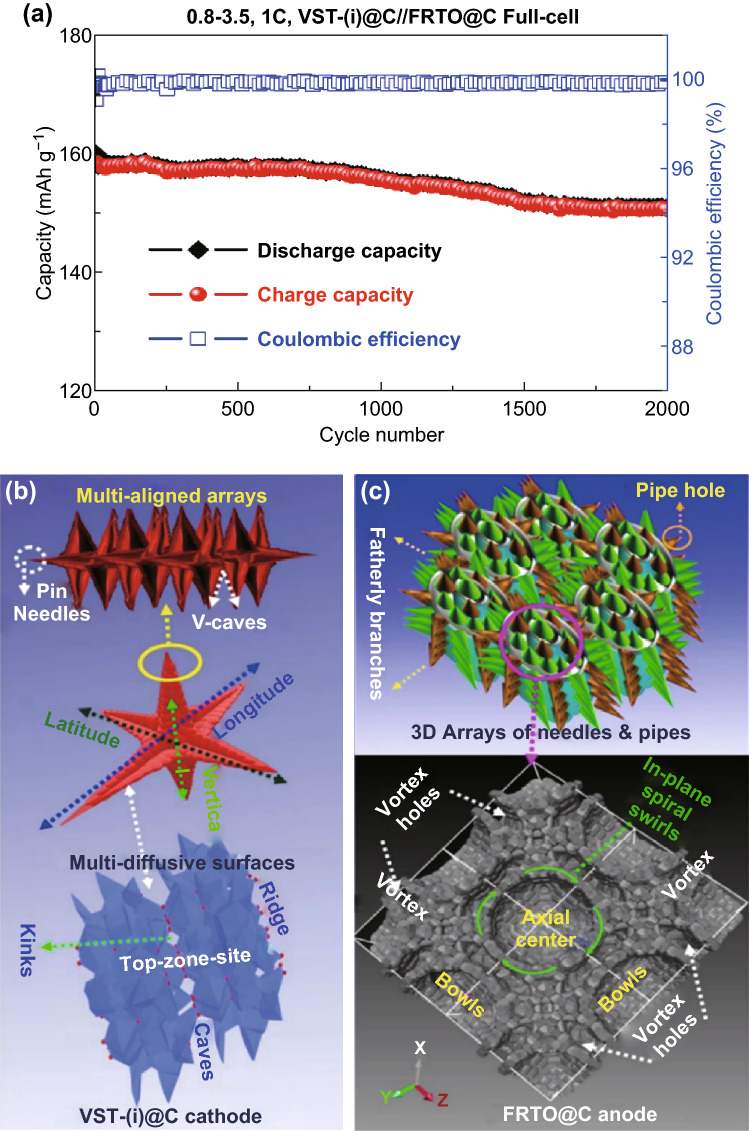
**a** Long-term cycling performance (stability) and Coulombic efficiency for a scalable full-cell VST-(i)@C//FRTO@C hierarchy at a rate of 1 C up to 2000 cycles and in the voltage range 0.8–3.5 V at room temperature. **b**, **c** 3D modeling projects of multi-directional along the vertical, lateral, and longitudinal axes of the super-hierarchical nano-architectures of VST@C cathode and FRTO@C anode electrodes. The retention of configuration in 3D-planes and modulate crystal structure surfaces (bottom) on the cathode and anode were evident. In-plane view projections of surface crystal modules recorded in a direction parallel to the upper atomic surfaces cathode/anode electrodes were investigated by DFT calculations after the multiple lithiation/delithiation (discharging/charging) cycling process

Figure 6a shows the proof-of-concept of the outstanding cycling performance, stability, and Coulombic efficiency of the 3D super-scalable model full-cell VST-(i)@C//FRTO@C at a rate of 1 C up to 2000 cycles and a voltage range of 0.8 to 3.5 V at room temperature. The full-scale LIB built-up VST-(i)@C//FRTO@C set exhibits a superb cycling stability and high energy density of 127 Wh kg^−1^ with an excellent durability (retaining approximately ~ 94.2% of its first discharge capacity at 160 mAh g^−1^ within 2000 cycles) and a 99.89% average Coulombic efficiency at a rate of 1 C at room temperature. For a higher cycle number, the VST-(i)@C//FRTO@C full-cell demonstrates ~ 100% of its Coulombic efficiency. The measurements also point toward the outstanding cycling performance, stability, and Coulombic efficiency of the 3D super-scalable model full-cell VST-(i)@C//FRTO@C built-up sets that satisfy the required energy density limit for EVs and scale-up commercial requirements. Our proposed LIB design and structures with outstanding electrochemical fulfillments hold much potential and can meet the requirements of large scale-up commercial LIBs and EVs in terms of energy density, power, specific capacity, and long-term life cycle compared with other reported LFPO cathode and TiO_2_ anode compositions, as shown in Table S5.

To explore the retention of 3D modeling projects along the vertical, lateral, and longitudinal axes of the super-hierarchical nano-architecture VST@C cathode and FRTO@C anode electrodes during cycling, we performed a set of DFT calculations to modulate the crystal structure surfaces (bottom) of the cathode and anode (Fig. 6b, c bottom) after around 2000 cycles of the lithiation/delithiation (discharging/charging) cycling process. The configurations of 3D planes and modulated crystal structure surfaces (bottom) of the cathode and anode were obviously retained. The in-plane-view projections of surface crystal modules recorded in the direction parallel to the upper atomic surface cathode/anode electrodes were investigated via DFT after a multiple lithiation/delithiation (discharging/charging) cycling process. In such a power hierarchy LIBs built-up set, the hotkey stability of the harmonious atomic-scale cathode/anode probably results in the assembly of surface LIB electrode podiums with flexible, multiple direction, building blocks and units. These multi-diffusive surface systems attained their uniformly directional movement/diffusion continuality throughout all dimensions and loops even after 2000 charge/discharge cycles. The power-hierarchy built-in full-cell-LIB electrode surface blocks have needles, caves, grooves, pipes, apex, edges, and vertex-ended surfaces for efficient floor plates, exterior vertical fins in multi-directional dimensions, and transport modules for achieving high-mobility electron/ion flows and transitions as well as high performance capacity. Furthermore, the in–out-plane of the upper zone surface of the open-multi-directional tower wings craving a dynamic environment and can accommodate seamless transition vacancy channels and tunnels (Fig. 6b, c bottom). This makes it a promising candidate for the next generation of high-power LIBs in terms of high discharge capacitance, high energy density and power, safety characteristics, and life cycle.

### Effect of Charge/Discharge Cycling on the Stability of Super-Hierarchical Cathode/Anode Building Blocks

**Fig. 7:**
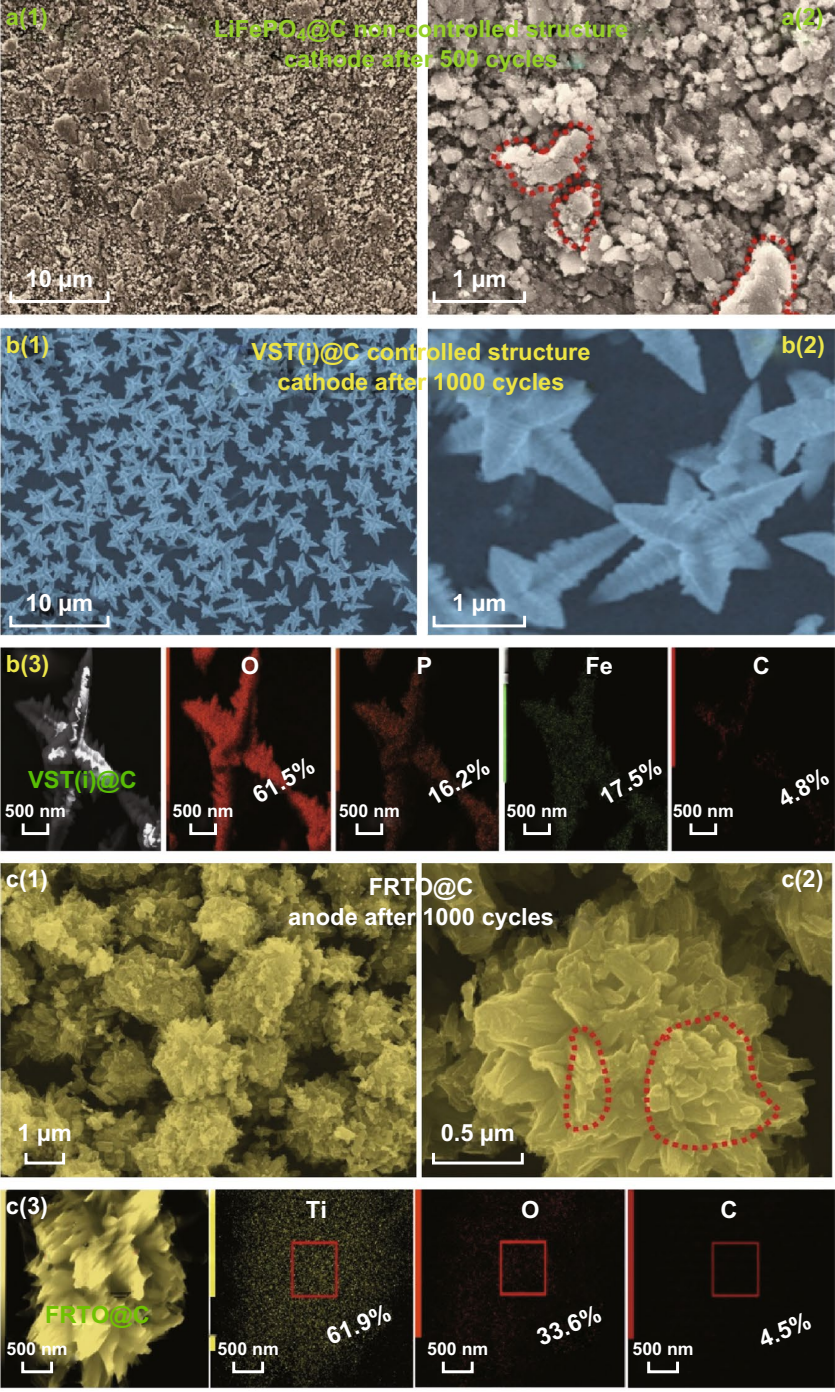
FE-SEM images with low- and high-magnifications of the LiFePO_4_ non-controlled structure (half-cell cathode) (**a**), super hierarchically shaped VST cathode (**b**), and the uniformly ordered FRTO@C anode (**c**) after long-term cycling at 1 C. The red dashed lines highlight the morphological changes via agglomeration or delocalization of the cathode/anode samples. EDS elemental mapping after 1000 cycles of the VST(i)@C cathode and FRTO@C anode in a full-scale model [**b**(3), **c**(3)]

To investigate the effect of multiple charge/discharge cycling on a super hierarchically shaped VST cathode, uniformly ordered FRTO@C anode, and non-controlled LFPO@C cathode, we recorded the FE-SEM microscopic patterns after 1000 cycles (Fig. 7). Figure 7a shows the FE-SEM images for a cycled sample of a non-controlled LiFePO_4_@C half-cell cathode at 1 C. The SEM patterns reveal the formation of large agglomerate particles due to cycling up to 500 cycles at 1 C (Fig. 7a-1, b). The instability of non-controlled LiFePO_4_@C structures may lead to a low electrochemical performance, as evidenced by their capacity, cycling stability, and capability results (Figs. S14, S16).

Figure 7b confirms the absence of any particular aging phenomenon of the VST(i)@C cathode sample after 1000 cycles of (VST(i)@C//FRTO@C) full-scale LIB at a current rate of 1 C. The microscopic images of the cycled cathode are similar to those of pristine VST(i)@C samples, as evidenced by Figs. 1a-1, a-2, and S4. The microscopic profile of VST@C confirms the excellent structure stability and outstanding electrochemical results, even after 1000 cycles, as seen in Fig. 4a–g. Figure 7c shows that, at first glance, no dramatic differences can be observed for the FRTO@C anode morphology after 1000 cycles of (VST(i)@C//FRTO@C) full-scale LIB at a current rate of 1 C. However, the surface potential reveals some irregular arrangements and delocalization in the feathery branches that are not observed in the original structure electrodes (Fig. 1j-1, j-2). Indeed, the FRTO@C anode after long-term 1 K cycling of the (VST(i)@C//FRTO@C) full-scale LIB at 1 C formed some agglomerates of its feathery prickly spines and dendritic fleshy needle-ended branches along its surface, as shown by the red dashed lines (Fig. S17c-2). Such structural damage of the cycled FRTO@C anode may be responsible for the full cell capacity fading over 1000 cycles (Fig. 6a).

Moreover, EDS elemental mapping after 1000 cycles of the VST(i)@C cathode and FRTO@C anode in the full-scale model is shown in Fig. 7b-3 and c-3. The elemental mapping compositions and distributions confirmed that there were no significant changes compared with the corresponding elemental mapping images of the cathode and anode prior to the cycling process, as shown in Fig. 1b, k, respectively. The EDS results revealed a nearly identical elemental distribution compared to the pristine electrode (Fig. 1b, k), even after 1000 cycles at a current rate of 1 C. This finding indicates the stability of the VST(i)@C//FRTO@C full cell during extended cycling. The retention of a high energy density may also open an avenue for the use of this cell in future EVs.

## Conclusions

As a mode of choice anode/cathode architectonics in LIB design, novel 3D power hierarchy LIB built-up sets for LIB-EVs applications with superb energy density, outstanding cyclability and Coulombic efficiency, superior durability, and excellent capacity retention were fabricated. This study is the first to evaluate the hotkeys of choice anode/cathode architectonics in terms of 3D super-scalable hierarchal models to create isosurface potential electrodes with mobile electronic movements, in-to-out interplay electron dominances, and well-defined coverage electron/charge cloud distributions. Our intensive theoretical/experiment sets based on the choice VST cathode architectonics modulated in powerful built-in LIB models indicated that the scalable VST-(i)@C-type architectonics dynamically provide effective diffusion gateways in multiple arrangement scales along the upper-, middle-, and lower-zone surface patterns and frontal edge/apex/central facets to achieve LIBs with excellent specific capacities, free pathway Li^+^ diffusions, facile lithiation and delithiation processes, high energy density, and long-term stability. Our proposed DFT surface analysis of the building blocks of a full-scale battery system indicate the potential effect of the choice anode/cathode electrode architectonics in terms of diversity, flexibility, and reliability on high-performance full-scale LIB built-up models. The power hierarchy VST-(i)@C//FTOR@C full-cell LIB built-up sets have long-term stability and an excellent capacity retention of 94.2%, with a 99.85% Coulombic efficiency for 2000 cycles at a rate of 1 C and high energy density of 127 Wh kg^−1^, thereby satisfying the energy density limits for the driving range of EVs and scale-up commercial requirements.

## Electronic supplementary material

Below is the link to the electronic supplementary material.

Supplementary material 1 (PDF 1832 kb)
