# Supplementary material for: Theoretical and Experimental Sets of Choice Anode/Cathode Architectonics for High-Performance Full-Scale LIB Built-up Models
Source: Nanomicro Lett. 2019 Oct 10;11:84. doi: 10.1007/s40820-019-0315-8 (PMC7770700; doi:10.1007/s40820-019-0315-8)
Supplement: Supplementary file 1 — Supplementary material 1 (PDF 1832 kb) [file 40820_2019_315_MOESM1_ESM.pdf]

Supporting Information for

Theoretical and Experimental Sets of Choice Anode/Cathode Architectonics for High-performance Full-scale LIB Built-up Models

H. Khalifa[1], S.A. El-Safty[1,]*, A. Reda[1], M.A. Shenashen[1], M.M. Selim[2], A. Elmarakbi[3], H.A. Metawa[4]

[1]National Institute for Materials Science (NIMS), Sengen 1-2-1, Tsukuba, Ibaraki 305-0047, Japan

[2]Department of Mathematics, Al-Aflaj College of Science and Human Studies, Prince Sattam Bin Abdulaziz University, Al-Aflaj 710-11912, Saudi Arabia

[3]Department of Mechanical & Construction Engineering, Faculty of Engineering and Environment, Northumbria University, Newcastle upon Tyne, NE1 8ST, UK

[4]Department of Physics, Faculty of Science, Damanhur University, Egypt

*Corresponding author. E-mail: sherif.elsafty@nims.go.jp (S.A. El-Safty)

S1 Half-cell and Full-scale LIB Designs for Electrochemical Performances of Power Hierarchy VST-(i-vii)@C Cathodes and FRTO@C Anodes

For generation of half-cell and full-scale LIB designs for electrochemical performances of power hierarchy VST-(i-vii)@C cathodes and FRTO@C anodes, all consumed chemicals have high analytical grade and used without further purification. Lithium hexafluoro-phosphate $LiPF_6$, carbon black and polyvinylidene fluoride (PVDF) are from Sigma–Aldrich Company, Ltd., USA. N-methyl-2-pyrrolidone (NMP) from Tokyo Chemical Industry (TCI) Company, Ltd., Tokyo, Japan. Electrochemical measurement of Li-ion intercalation was performed using CR2032 coin-cells that assembled in a glovebox under pure Ar-gas, as shown in Fig. S1.

For fabrication of VST(i-vii)@C cathodes, FRTO@C anode as half-cell working electrodes, we used lithium foil used as reference and counter electrode. VST(i)@C//FRTO@C (cathode//anode) full-cell LIBs were successfully fabricated for electrochemical measurements. In order to fabricate the cells, the liquid electrolyte prepared as a solution of $LiPF_6$ (1 M) conductive salt in ethylene carbonate/diethyl carbonate (EC:DEC) (1:1 v/v). The working electrodes were prepared by mixing each active material of FRTO@C anode composite and VST(i-vii)@C cathode materials with conductive carbon black, followed by the addition polyvinylidene fluoride (PVDF) as a binder in a weight ratio of 75:15:10 and dissolved in N-methyl-2-pyrrolidone (NMP) under stirring for 1 h.

The full-cell LIB is fabricated under optimized mass loading (balancing) – (N/P) ratio (where N = negative anode electrode capacity; and P = positive cathode electrode capacity in mAh**)**. The full-scale cathode//anode stacked layers pouch LIB-model, mass loading, areal capacity and volumetric energy density were examined. The mass loading is 13 and 6.9 mg cm^{-2} of cathode and anode active materials. Moreover, the areal discharge capacity is 1.13 and 1.19 Ah cm^{-2} for the cathode and anode electrodes, respectively. The general balancing between cathode and anode based on the assumption that discharge specific capacity (in Ah) is equal for negative and positive electrode giving the (N:P)Cap capacity ratio = 1.05:1. This latter

(N:P)Cap value is used in order to optimal trade–off between the better safety (oversizing of negative electrode capacity, and then (N:P)Cap capacity ratio = >1:1). The optimum specific energy may be obtained at equal capacities of negative and positive electrode, and then (N:P)Cap capacity ratio can be =1:1) for proposed stacked $LiFePO_4$//TiO_2 pouch LIB-model.

The slurries were casted onto aluminium foil (10 µm thickness) for LFPO@C cathodes and copper foil (8 µm thickness) for TiO_2 anodes and then dried in a vacuum oven at 80 °C for 12 h. The dried thick film electrode was pressed between twin rollers to enhance its packing density, reduce the porosity of the film and ensure intimate contact of the active material and the current collector. A microporous polymer separator is supplied from Celgard 2400TM membrane, Hoechst Celanese Corporation, Charlotte, North Carolina, USA).

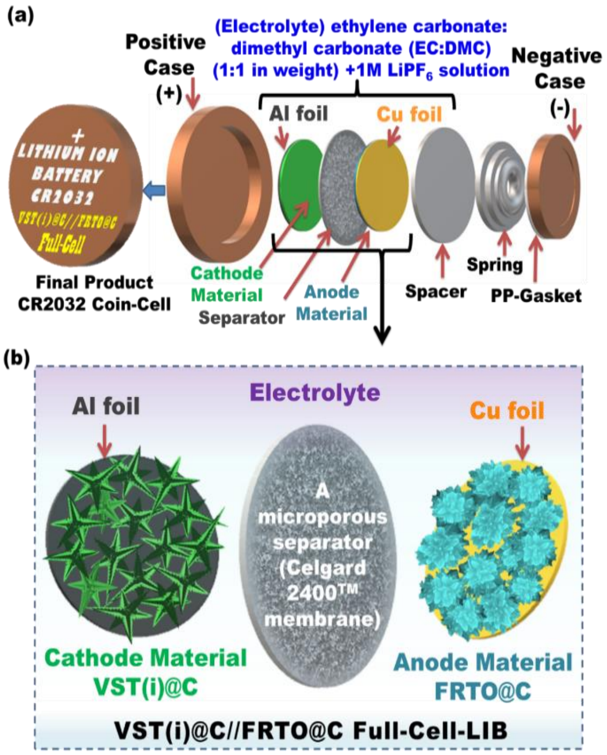

Fig. S1 a Illustration of a CR2032-type coin cell assembly. **b** Schematic design of formulation of FRTO@C (anode) and VST(i)@C (cathode) electrodes designated in coin-cell CR2032-type full-LIB

To prepare 2032-coin cells, circular electrodes with diameters of 16 mm were used for Li foil and working electrodes. A separator with 20mm diameters was punched for further use. Crimper machine for the CR20XX series coin cells utilized to press our 2032-coin cells inside the glove box under Ar. The designated VST(i)@C//FRTO@C cathode//anode 2032-full-cell LIBs designated for electrochemical measurements (Fig. S1). Moreover, full-scale VST(i)@C//FRTO@C cathode//anode stacked layers pouch LIB-model are designed for further use. The prepared batteries were left for 24 h prior to be tested in order to guarantee total intake of the electrolyte solution by electrodes. Galvanostatic charge/discharge characteristic were measured using multichannel battery system (LAND CT2001A, Wuhan, China). Cyclic voltammetry (CV) measurements of the cells were tested using (CHI 660c electrochemical workstation). Electrochemical impedance spectroscopy (EIS) were performed using (Zennium/ZAHNER-Elektrik GmbH & CoKG, controlled by Thales Z-3.0 software – frequency range from 0.1 Hz to 1 MHz). All the electrochemical measurements were done at room temperature (30 °C).

Table S1 Mass fraction of individual components used in the pouch full-cell

Pouch cell components	Mass (%)	Mass (g)	Active Material
**Cathode**	43.4	2.61	75% $LiFePO_4$ cathode active material (1.96 g); 15% carbon black (0.39 g); 10% PVDF as a binder (0.26 g)
**Anode**	22	1.32	75% TiO_2 anode active material (0.99 g); 15% carbon black (0.2 g); 10% PVDF as a binder (0.13 g)
**Al foil collector**	3.6	0.21	
**Cu foil**	7	0.42	
**Separator**	3	0.18	
**Electrolyte**	13	0.78	
**Packaging**	8	0.48	
**total**	100	6	

S2 Characterization Techniques and Materials

All consumed chemicals have high analytical grade therefore they have been used as it is without further purification. Lithium Acetate dihydrate ($CH_3COOLi{\cdot}2H_2O$), Iron III nitrate nonhydrate ($Fe(NO_3)_3{\cdot}9H_2O$), Hydrogen peroxide solution (H_2O_2) and ethanol(C_2H_5OH) were purchased from Sigma–Aldrich Company, Ltd., USA. Phosphoric acid (H_3PO_4) from Tokyo Chemical Industry (TCI) Company, Ltd., Tokyo, Japan. Ethylene Glycol ($C_2H_6O_2$), Hydrochloric Acid (HCl)(2mol/L) and Titanium (IV)-oxysulfate (Titanoxysulfat-$TiO(SO_4){\cdot}xH_2O$)from Nacali Tesque Company, Ltd., Kyoto, Japan. All chemicals were added in stoichiometric ratios, TiO_2 and $LiFePO_4$ compositions were synthesized by the hydrothermal method. Carbon coating process was carried by microwave irradiation technique.

X-ray diffraction (XRD) characterization of power hierarchy VST-(i-vii)@C cathodes and FRTO@C anodes was performed to investigate the crystallographic information of the annealed samples using a 18 kW diffractometer (Bruker D8 Advance X-ray diffractometer) at scan rate of 10° min^{-1} with CuKα-X-radiation (λ = 1.54178 Å) at 30 kV and 10 mA (Figs. 2 and S10).

Field-emission scanning electron microscopy (FE-SEM) and transmission electron microscopy (TEM) of power hierarchy VST-(i-vii)@C cathodes and FRTO@C anodes were used to investigate the details of the structure. The morphologies of our samples were investigated by Field emission-type Scanning electron microscope FE-SEM (Jeol JSM-Model 7000F, JEOL Ltd) at 20 kV. This SEM is equipped with a Schottky (thermal type) field emission electron gun. Analysis material was fixed onto the FE-SEM stage using carbon tape before insertion into the FE-SEM chamber. The ion sputter (Hitachi E-1030) was used to deposit thin-layered Pt films on electrodes at 25 °C (Figs. 1 and S4-S9, Scheme 2).

High-resolution transmission electron microscopy (HRTEM) images, electron diffraction (ED), scanning transmission electron microscopy (STEM) of power hierarchy VST-(i-vii)@C cathodes and FRTO@C anodes were performed at atomic-level imaging, structural and chemical analysis field emission-type TEM (JEM-ARM200F), as shown in Fig. 2. The

accelerating voltage can be adjusted to 80, 120, or 200 kV, depending on the specimen material and the purpose of observation. Energy-dispersive X-ray spectroscopy (STEM-EDS) was carried out during TEM measurements with high-resolution elemental mapping for of power hierarchy VST-(i-vii)@C cathodes and FRTO@C anodes to investigate the chemical contents of the nanostructures by using a 200 kV TEM (JEOL 2100F, JEOL Ltd) field emission-type transmission electron gun microscope. The samples were dispersed in ethanol solution using an ultrasonic radiation, and then dropped on a copper mesh and vacuum dried for 20 min prior to inserting the samples into the HR-TEM and STEM-EDS columns. Prior to microscopic investigation and analysis, the sample powders were dispersed in ethanol solution by means of ultrasonic-radiation, then one drop is added to copper mesh surface and vacuum dried for 20 min before used in HR-TEM and STEM-EDS columns.

The surface properties of VST@C and FRTO fabrics were estimated by N_2 adsorption–desorption isotherms at 77 K using a BELSORP36 analyzer (JP. BEL Co., Ltd.). The samples were thermally treated at 200 °C for at least 6 h under N_2 atmosphere. The specific surface area (SBET) was calculated using the Brunauer–Emmett–Teller (BET) method with multipoint adsorption data from the linear section of the N_2 adsorption isotherm. The pore size distribution was determined using nonlocal DFT (NLDFT). Figure 2i that type IV isotherm with an H_2 hysteresis loop is dominant for all tested VST-(i-vii) samples, which confirms its mesoporous structure. Figure 2j shows the pore size distribution curves with calculated pore diameter of our tested samples. The S_{BET} surface area decreases in this order: 330.3 < 272.7< 208.5< 112.1< 89.7< 24.9 <23 m^2 g^{-1} for VST-(i), (ii), (iii), (iv), (v), (vi), and (vii) samples, respectively. The pore size diameters for different structures VST-(i), (ii), (iii), (iv), (v), (vi), and (vii) particles were in the range 13.1- 52.1 nm, see insert in Fig. 2. This finding indicates that the super-scale hierarchal VST@C cathodes oriented with open-end vestibule corridors of VST-(i) and (ii), and with dense floor-on-floor building blocks VST-(iii, iv, v, vi, and vii) morphologies affected surface parameters of electrodes.

The weight content loss of power hierarchy VST-(i-vii)@C cathodes were determined from the weight loss curve measured under simulated air atmosphere on thermo-gravimetric and differential scanning calorimetry (TG/DSC) instrument TG/DSC-60 (Shimadzu, Japan) with a heating rate of 10 °C min^{-1}.

X-ray photoelectron spectroscopy (XPS) analysis (0-1400eV) of power hierarchy VST-(i-vii)@C cathodes and FRTO@C anodes was conducted on a PHI Quantera SXM (ULVAC-PHI) instrument (Perkin–Elmer Co., USA) equipped with Al K_α radiation as an X-ray source for excitation (1.5×0.1 mm^2, 15 kV, 50 W) under a pressure of 4×10^{-8} Pa. Raman spectroscopy (HR Micro Raman spectrometer, Horiba, Jobin Yvon) was conducted using an Ar ion laser at 633 nm. A charge coupled device (CCD) camera detection system and the LabSpec-3.01C software package were used for data acquisition and analysis, respectively.

Raman spectroscopy of power hierarchy VST-(i-vii)@C cathodes and FRTO@C anodes was pplied to determine the chemical structures of both anode and cathode in full-scale LIBs. To ensure the accuracy and precision of the Raman spectra, 10 scans of 5 s from (500-2000 cm^{-1}) were recorded. The chemical compositions of power hierarchy VST-(i-vii)@C cathodes and FRTO@C anodes were analyzed by Fourier transform infrared spectroscopy (ATR-FTIR, Spectrum 100, Perkin-Elme, Inc., USA) (Figs. S11-S14).

Density functional theory (DFT) and electrostatic potential and electron maps (ESP-EM) of power hierarchy VST-(i-vii)@C cathodes and FRTO@C anodes can be studied according to DMol3 of BIOVIA Dassault systems. The Perdew–Burke–Ernzerhof (PBE) formalism was also used for exchange-correlation energy function [43]. DFT was applied to determine the

electrostatic potential (ESP) distribution along the TiO_2@C, and $LiFePO_4$@C (anode and cathode) electrode surfaces designated in closely-packed flower agave rosettes (FRTO) and in vertical-star-tower (VST) building blocks. The ESP sowed the Coulomb interaction per unit charge of active site at position in space. 3D modeling objects were recorder according to the physical quantitative survey at each single isosurface point as shown from the representative surface electron/charge map (EM). Typically, the electron densities along isosurfaces were colored according to ESP intensities (ESPI) of the crystal lattice of super-scalable hierarchal models of FRTO anode and VST cathode, in which the surface charges of lattice would be mapped "so called contour". The 3D slab and super-scalable hierarchal models of FRTO anode and VST cathode was constructed according to multiple atomic building block layers. ESP was investigated over the range of −0.06 to +0.6 eV as shown in recorded designs (Scheme 2),

S3 Mechanistic Formation of Anode/Cathode Architectonics

The mounts of uniformly cathodic morphologies with flexible, multiple blocks and units at high-end tower roofs VST-(i), VST-(ii), VST-(iii), VST-(iv), VST-(v), VST-(vi), and VST-(vii) architectonics can be tailored by varying the ratios of the Et/EG solution. The additives of Et/EG mixture ratios (100%:0), (83.33%:16.67%), (66.67%:33.33%), (50%:50%), (33.33%:66.67%), (16.67%:83.33%), and (0:100%) allow the preparation of VST-(i), VST-(ii), VST-(iii), VST-(iv), VST-(v), VST-(vi), and VST-(vii) buildings during the hydrothermal treatment, respectively. This phenomenon can be attributed to several reasons. For instance, H_3PO_4 releases PO_4^{3-} anions that produces FeH_2PO_4 as the first nucleation seed. Meanwhile, the OH^- active ions released from the addition of Et/EG can coordinate and build frameworks with Fe^{3+}.

The key components that control the super-scalable model include the OH^- amount, which can change the reaction kinetics and influence the nucleation and growth pathways of LFPO crystals as well as the formation of the VST configuration in a block/stacked or open morphology. The high EG concentration (i.e., 100%) facilitates the spread and diffusion of OH^- ions in the vessel and subsequently transforms the EG into a directing orientation agent for reducing the interaction surface potential, which may lead to the arrangement of a high-rise mixed-complex building in a tip-to-tip configuration along an axial center at its core to occupy the framework geometry in multiple directions. The intensive c-axis orientation of superstructured surfaces lead to block/stacked building blocks on each floor (layer) of the most complex 3D-hierarchical VST (vii) structure. OH^- ions are quantitatively added to the LFPO composition domain by increasing the Et/EG ratio up to (Et, 100%), which changes the densely compact building with full 3D open wings and the directions of the VST modulate superstructure VST(i). The addition of 100% EG to the mixture synthesis domains will significantly change the open grooves, convex-up planar needle wings, and well-oriented VST-(i) tower projection to VST-(vii) fully stacked coverage flanks at the in-out-plane of the upper zone surface of open-, multi-directional tower wings (Fig. 1a-i). Among all VST super-star-towers, the VTS-(i) design can be considered a free manifold nucleation of LFPO crystals in well-designed orientations.

Another key factor in the formation of the VST-(i) to VST-(vii) designs is the time-dependent, drop-wise insertion of OH^- (i.e., Et/EG mixture), which is dominated by a well-controlled layer-by-layer growth fabrication. The slow kinetic-binding rate mode of species can provide a sufficient relaxation time to define the formation of the open-space building blocks in specific 3D hierarchy and multi-orientations, such as VST-(i), compared with the formation time rate of unorganized and condensed structure VST-(vii) blocks.

In general, chemically controlling the addition of high EG amounts in composition domains can increase the number of VST block layers and cover the outer entrances, vacancies, grooves, and wings of the entire building block, thereby diminishing all possible free-movement electron, gateways, and diffusion pathways (Figs. S4–S9). The drop-wise addition of H_2O_2 to $TiO(SO_4)$ also contributes to the formation of a flower sphere-like vase with solitary succulents and feathery prickly spines. The time-dependent treatment also leads to the formulation of agave rosettes with fleshy needle-ended branches (Fig. 1j–k).

S4 Vertical-star-tower Building Blocks of VST-(i)@C Cathode

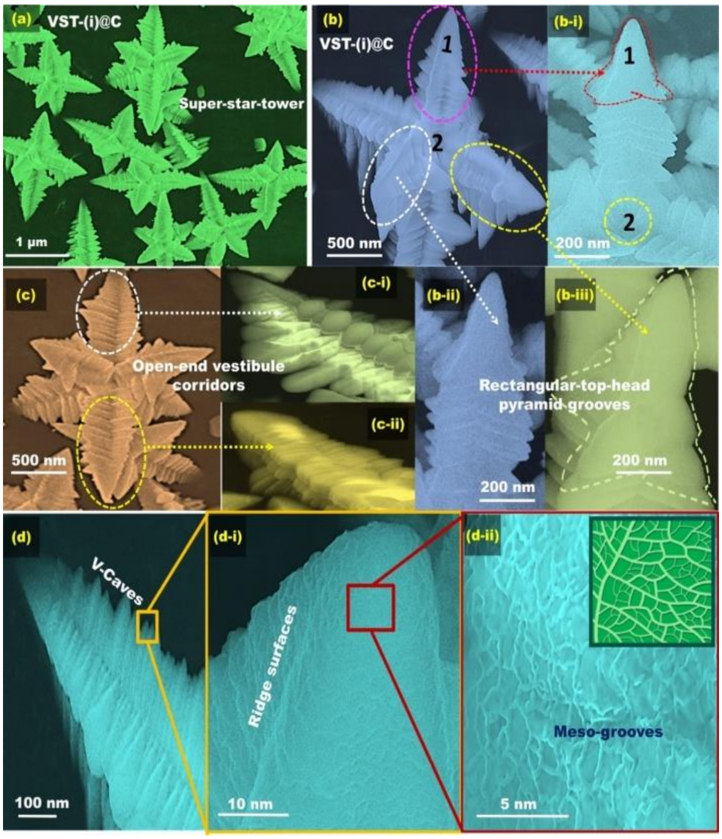

Fig. S2 a-d Low and high-resolution FE-SEM images of hierarchal olivine super vertical-star-tower building blocks with axial-pillar and prism tip labeled as VST(i)@C-cathode. **b-i (inset-1)** Prism tip (tower top) and **(inset-2)** of tower core. **c** Longitudinal axis of VST-(i)@C showing scalable architectures dynamically provide effective diffusion gateways in multiple arrangement scales along upper, middle and lower-zone - surface patterns. **d** Tower top-view objects showing the wing details of VST-(i)@C included the zigzag main corridors with rooms on both sides, **(d-i)** enlarged part of tip of a room, **(d-ii)** High magnification of the zigzag tracks multi-meso-micro diffusive surface sites for Li^+-ions with similar structure shown in **(inset-d-ii)** as of reticulate structure with smaller veins forming a network

Topography and morphology of samples were investigated by FE-SEM. The FE-SEM image can be shown in Figs. S2-S7. FE-SEM microscope shed light about 3D super-scalable hierarchal models of vertical-star-tower building blocks for VST-(i-vii)@C cathodes and closely-packed flower agave rosettes FRTO@C anodes (Figs. S2-S7). In addition, energy-dispersive X-ray spectroscopy (STEM-EDS) was performed with high-resolution elemental mapping to investigate composition distribution of the composite chemical contents along the nanostructures morphological hierarchy VST-(ii-vii)@C, as shown in Figs. S3e, S4d, S5d, S6c, and S7g, respectively.

S5 Vertical-star-tower Building Blocks of VST-(ii)@C Cathode

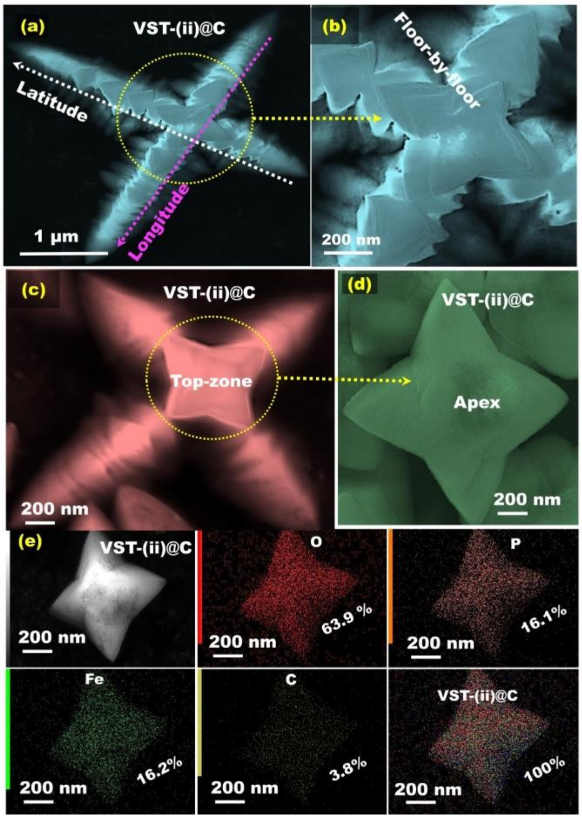

Fig. S3 a-d Low and high-magnification FE-SEM images of hierarchal olivine complex wings star shape (VST-(ii)@C). **e** FE-SEM bright filed image and elemental mapping of VST-(ii)@C

S6 Vertical-star-tower Building Blocks of VST-(iii)@C Cathode

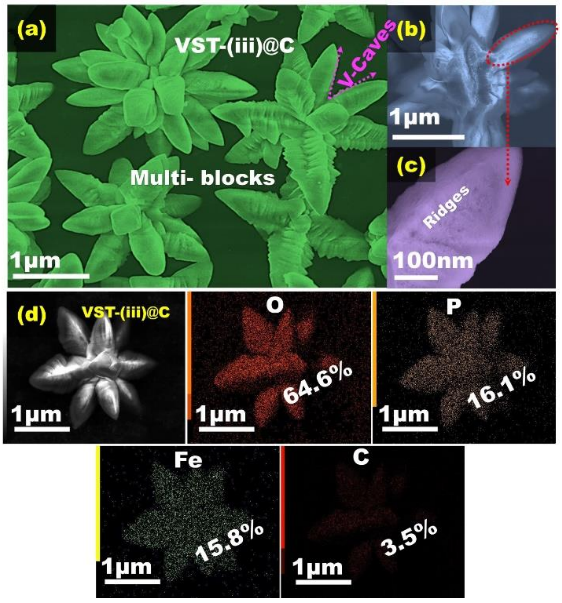

Fig. S4 a-c Low- and high-resolution FE-SEM images of hierarchal multi-blocks VST(iii)@C material. **c** High-resolution FE-SEM image shows the macro/mesoporous structure on the surface of arm of VST-(iii)@C. **d** FE-SEM image and elemental mapping of VST-(iii)@C

S7 Vertical-star-tower Building Blocks of VST-(iv)@C Cathode

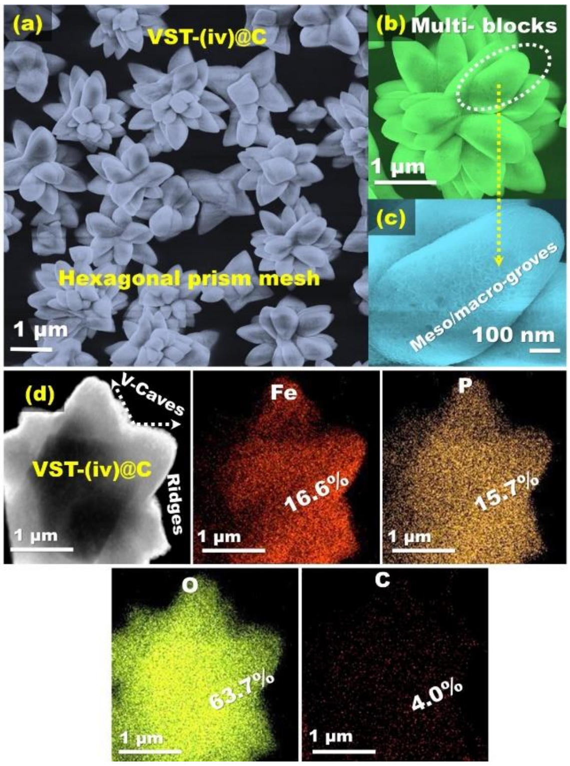

Fig. S5 a-c Low and high-magnification FE-SEM images of hierarchal olivine VST-(iv)@C. **c** High-resolution FE-SEM image shows the macro/meso porous structure on the surface of arm direction of VST-(iv)@C. **d** FE-SEM image and elemental mapping of VST-(iv)@C

S8 Vertical-star-tower Building Blocks of VST-(v)@C Cathode

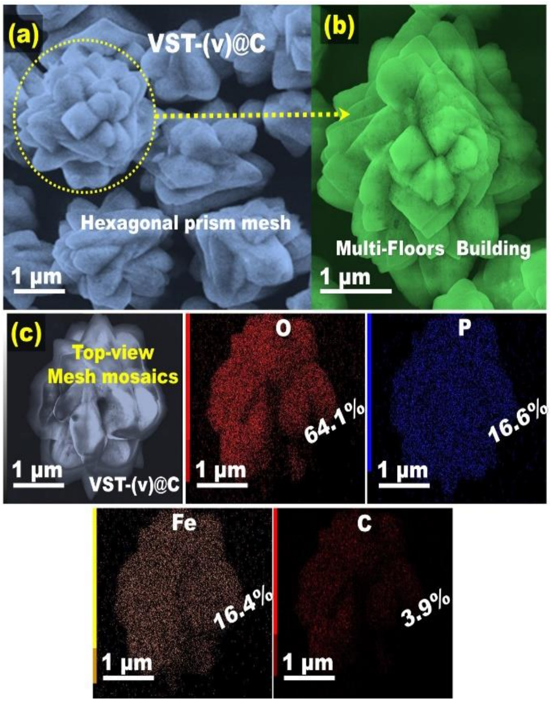

Fig. S6 a, b Low and high-resolution FE-SEM images of hierarchal olivine VST-(v)@C. **c** FE-SEM mesh mosaics and elemental mapping of VST-(v)@C

S9 Vertical-star-tower Blocks of VST-(vi)@C and VST-(vii)@C Cathodes

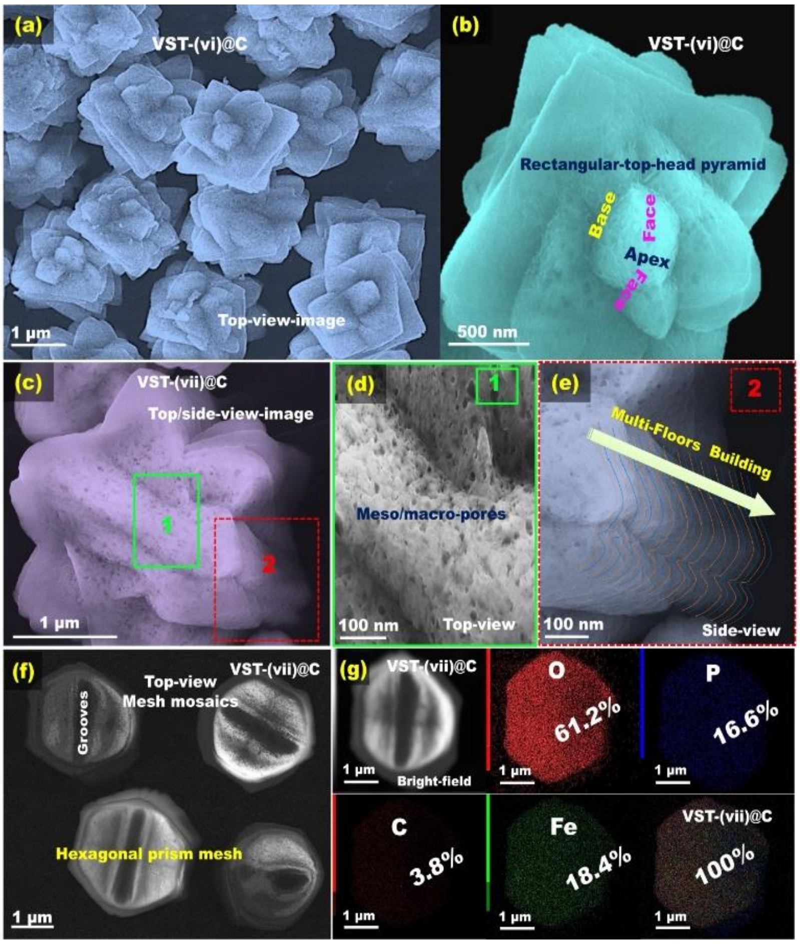

Fig. S7 a Low and **b** high-magnification FE-SEM images of hierarchal olivine VST(vi)@C. **c** high-magnification FE-SEM images of hierarchal olivine VST(vii)@C. **d** high- magnification FE-SEM image of area noted by 1 as inset of **c** showing the macro/meso porous structure on the surface of VST(vii)@C. **e** high- magnification FE-SEM image of area noted by 2 as inset of **c** shows the direction of multi- floor-building layers of VST(vii)@C from bottom to top. **f** High-magnification FE-SEM images of meh mosaics VST-(vii)@C. **g** FE-SEM image and elemental mapping of VST-(vii)@C

S10 Crystal Structures of Vertical-star-tower Blocks of VST Cathodes

X-ray diffraction (XRD) is used to examine the change in the phase transition and crystal structures of the annealed super-scale hierarchal VST@C cathode synthesized with open-end vestibule corridors of VST-(i) and (ii), and with floor-on-floor building blocks VST-(iii, iv, v, vi and vii) morphologies. Structural properties of prepared VST-(i, ii, iii, iv, v, vi and vii) cathode-particles were evaluated by X-ray diffraction (XRD) characterizations (Fig. S8). X-ray patterns are similar for all prepared LFPO based super-scale hierarchal VST@C cathode with open-end vestibule corridors of VST-(i) and (ii), and with floor-on-floor building blocks VST-(iii, iv, v, vi and vii) morphologies, Figs. 2a and S8. All diffraction peaks are indexed to the well-defined pure orthorhombic olivine-type structure of LFPO, space group of Pnma, which are agreed with the reported values (JCPDS No. 83-2092) [64]. The diffraction patterns for all samples showed no evidence for impurity structures (such as Li_3PO_4, $Li_3Fe_2(PO_4)_3$, conductive FeP or $Li_4P_2O_7$). These results indicate the stability of the LFPO orthorhombic olivine-types, despite the change in the different in the (VST) building blocks, multi-

directional orientation components, and isosurface potential changes withing fabricated VST morphologies. The obtained by Rietveld refinement results (a = 9.963 Å, b = 6.078 Å, and c = 4.678 Å, with an acceptable R_{wp} value of 1.238 and unit volume = 286.95Å^3), which are all in good agreement with the literature values (JCPDS No. 83-2092) [26, 45, 64–66]. Furthermore, the X-ray diffraction pattern Fig. 2b for flower like morphology TiO_2 as anode material (FRTO) agree with standard pattern of the tetragonal pure rutile structure TiO_2 (JCPDS No. 01-076-1939).

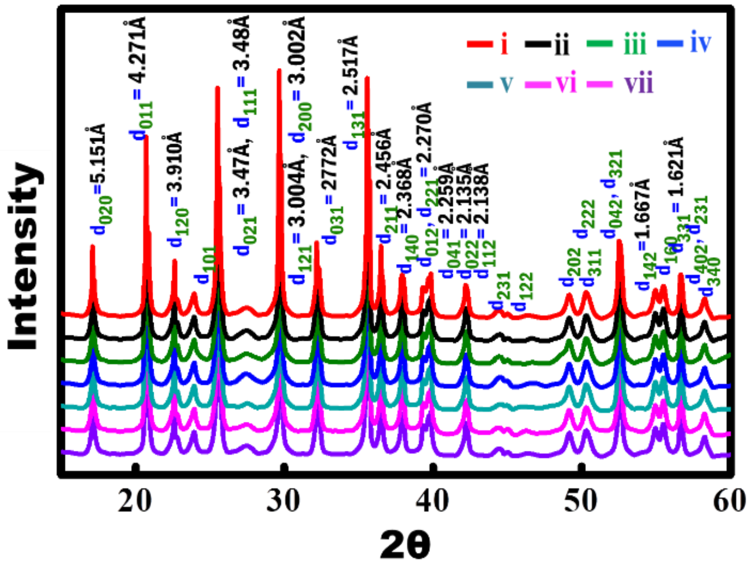

Fig. S8 XRD patterns of the as-synthesized super-scale hierarchal VST@C cathode with open-end vestibule corridors of VST-(i) and (ii), and with floor-on-floor building blocks VST-(iii, iv, v, vi and vii) morphologies

S11 Thermal Stability of Vertical-star-tower Blocks of VST Cathodes

The effect of super-scale hierarchal VST@C cathode with open-end vestibule corridors of VST-(i) and (ii), and with floor-on-floor building blocks VST-(iii, iv, v, vi and vii) morphologies on the thermal stability of cathode electrode was investigated by using thermogravimetric analysis TG and differential thermal analysis (DTA) measurements. Thermal stability of super-scale hierarchal VST@C cathode and loss in carbon content of VST-(i, ii, iii, iv, v, vi and vii)@C composites were investigated through thermo-gravimetric and differential scanning calorimetry (TG/DSC) method using TG-60 (Shimadzu, Japan) instrument under a continuous flow of air with a heating rate of 5 °C min^{-1} from 20 to 900 °C as shown in Fig. S9.

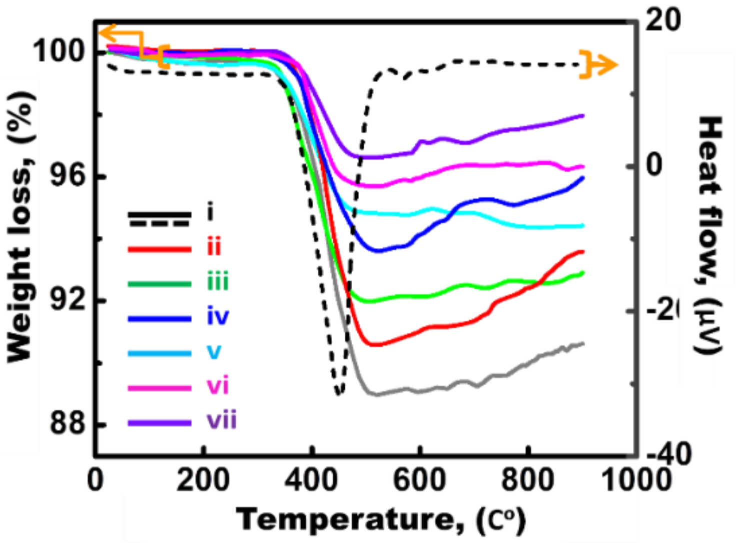

Fig. S9 TG and DTA curves of the super-scale hierarchal VST@C cathode with open-end vestibule corridors of VST-(i) and (ii), and with floor-on-floor building blocks VST-(iii, iv, v, vi and vii) morphologies. Differential scanning calorimetry (DSC) was measured for VST-i @C cathode.

TG curves of the VST-(i, ii, iii, iv, v, vi and vii)@C cathodic composites. No significant changes in TG/DSC profiles were observed for different super-scale hierarchal VST@C cathodes synthesized with open-end vestibule corridors of VST-(i) and (ii), and with floor-on-floor building blocks VST-(iii, iv, v, vi and vii) morphologies. Three discrete regions of weight loss or heat transfer of super-scale hierarchal VST@C cathodes synthesized with open-end vestibule corridors of VST-(i) and (ii), and with floor-on-floor building blocks VST-(iii, iv, v, vi and vii) morphologies were found. The first insignificant weight loss or heat transfer region below 350 °C, is basically produced by releasing of absorbed water and chemisorbed crystal water (desorption of water). The second region from 350 to 550 °C showed significant changes. These thermal effects in weight loss or heat transfer of present samples become significant. It is important to note that carbon coated samples may lead to offer high significant weight loss or heat transfer in the super-scale frameworks indicated the endothermic effects, which are mainly due to pyrolysis of the non-polymeric organic compounds such as glucose (c-sources) as shell-dressers of cathodic VST@C samples. The third weight loss or heat transfer region above 600 °C showed an insignificant change of weight loss or heat transfer due to the stability of crystal structures and atomic-scale frameworks of VST@C samples even when the treated temperature is further increased to 900 °C. At above 600 °C, exothermic or endothermic peaks are not not discovered in DSC curves and the mass of the sample is not changed, indicating that the oxidization reactions of VST-(i-vii)@C cathode composites were terminated.

This finding of TG /DTA measurement offer key clues on the fabrication of stable electrodes in full-scale LIBs as follows:

(i) The appropriate calcination temperature to form stable organic-inorganic frameworks is 600 °C;
(ii) Thermal stability of super-scale hierarchal VST@C cathodes;
(iii) Negligible effect of the morphological shapes of VST@C synthesized with open-end vestibule corridors of VST-(i) and (ii), and with floor-on-floor building blocks VST-(iii, iv, v, vi and vii) morphologies on thermal stability of the electrodes.

S12 Chemical Structure of Vertical-star-tower Blocks of VST Cathodes

The chemical compositions and stability of atomic-scale framework structures of the super-scale hierarchal VST@C cathodes synthesized with open-end vestibule corridors of VST-(i) and (ii), and with floor-on-floor building blocks VST-(iii, iv, v, vi and vii) morphologies were investigated by Fourier transform infrared spectroscopy, FTIR. FTIR analysis may shed lights of the effect of the morphological shapes in the composition domains of anisotropic, heterogeneous composites.

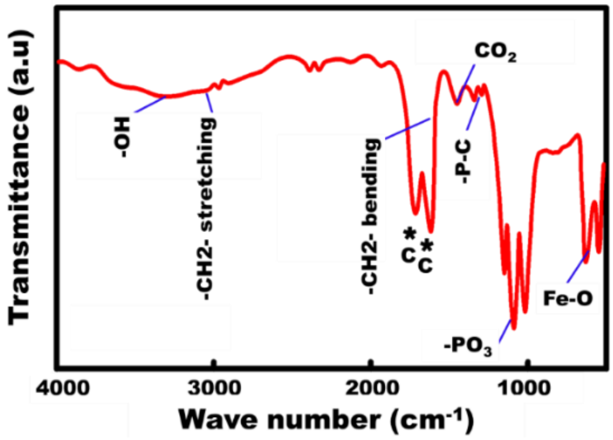

Fig. S10 FT-IR spectra of super-scale hierarchal VST@C cathode with open-end vestibule corridors of VST-(i) and (ii), and with floor-on-floor biulding blocks VST-(iii, iv, v, vi and vii) morphologies

Fourier transform infrared (FT-IR) spectra were performed for VST@C samples as shown in Fig. S10. FT-IR spectra of VST@C exhibit a peak at 578 cm^{-1}, small intense peak around 1440 cm^{-1} and peak at 3250 cm^{-1} that attributed to Fe−O, P−C and C=H2 stretching vibrations, respectively, in our samples. Also, strong band centered at 1090 cm^{-1} is ascribed to −CPO_3 group tetrahedral stretching vibration. Moreover, the weak peak at 1536 cm^{-1} is due to C=H2 bending vibration. Therefore, FT-IR result ensure the present of FeP framework. The C-shell dressed the VST exhibit characteristic peaks (Fig. S10, star marks), which indicate the organic-inorganic binding during the formation of the VST@C hierarchy. The FTIR spectra at 1615 and 1718 cm^{-1} suggests the existence of C=O stretching. It is important to note that FTIR showed no significant change in the peak signals with changes in the VST morphological VST-(i)-to-(vii) shapes. This finding indicates the stability of chemical compositions and stability of atomic-scale framework structures of the super-scale hierarchal VST@C cathodes synthesized with open-end vestibule corridors of VST-(i) and (ii), and with floor-on-floor building blocks VST-(iii, iv, v, vi and vii) morphologies

S13 Chemical Bonding and Composition of Vertical-star-tower Blocks of VST Cathodes

Raman spectroscopy is a powerful tool allows revealing of surface composition and chemical bonding along anisotropic and heterogeneous multi-components. Raman spectroscopy results for all VST@C composites are shown in Fig. S11. The bands at 640.0 and 939.6 cm^{-1} indicate the symmetric mode of Fe_3O_4 and PO_4^{3-}groups in VST@C composites. Two peaks at 1350.73 and 1582.45 cm^{-1} are shown, attributing to D and G bands of carbon bonding in VST@C, respectively. The C-D-peak matched with disordered carbon binding of highly defective graphite. In turn, the C-G- peak is related to (graphite, in-plane vibrations with E_{2g} symmetry). Therefore, according to Raman and FTIR analysis, the finding confirms the thin-loading of

carbon shell wrapped surface of VST@C. The carbon shell is cross-linked via C=C, C=N, C=O bonds to the $LiFePO_4$ frameworks.

Together, first, this Raman spectroscopic finding indicates the stable molecular binding of C-shell bumps wrapped inorganic LFPO frameworks and spatial distributions of carbon dots along the LFPO chemical compositions without dislocation or disturbance in the atomic-scale crystalline framework structures of the super-scale hierarchal VST@C cathodes and FRTO@C anodes. Second, there is no significant change in D and G bands of the carbon-type bonding along $LiFePO_4$ frameworks with 3D super-scalable model structures that synthesized with confinement open-end vestibule corridors of VST-(i) and (ii), and with dense stacking of layer-to-layer building blocks VST-(iii, iv, v, vi and vii) morphologies. Third, the prominent binding pairs between carbon and inorganic frameworks of TiO_2 anode and $LiFePO_4$ cathode may attain the super-scale molecular dimensionality and directionality along laterally, vertically, and longitudinally convex-up planar tubular pipes and grooves and at in-out-plane of the upper zone surfaces of TiO_2 anode and $LiFePO_4$ cathode designated in FRTO agave rosettes and vertical super-star-building block towers, respectively. Finally, the dynamic electron movements along super-scalable architectures, and anisotropic heterogeneity components would provide surface with effective diffusion gateways for excellent specific capacities, facile charge–discharge rates, high-energy- density, and long-timescale stability LIBs.

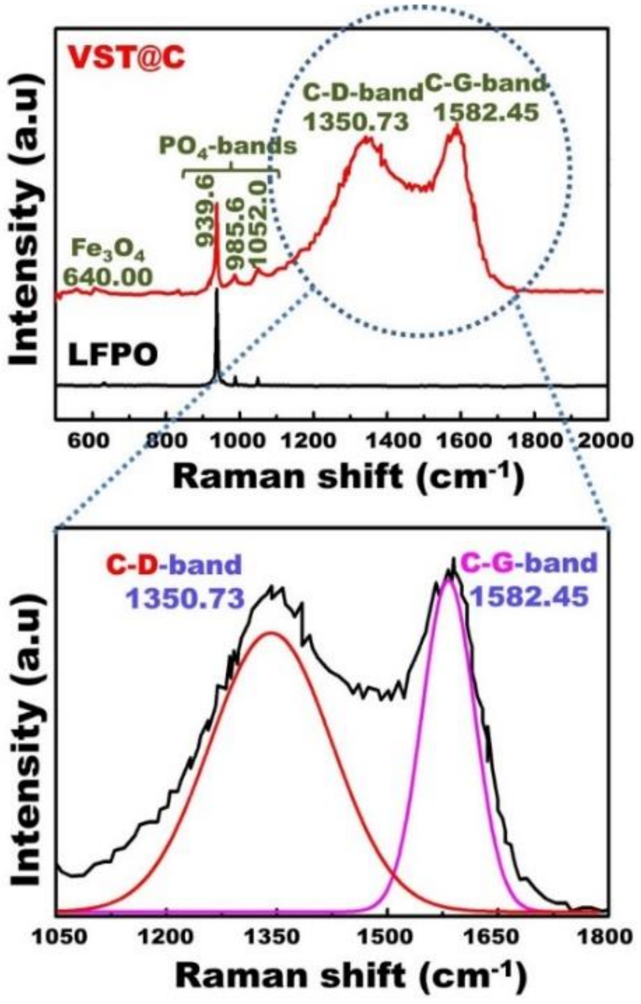

Fig. S11 Raman spectra of VST@C cathode with $LiFePO_4$@C composites

S14 Surface Binding Energy and Exposure Active-surface Sites in the Chemical Components

X-ray photoelectron spectrometry (XPS) shows evidence of the composition domains, oxidation states of active sites and binding surface energy along exposure top-zone surface sites of heterogeneous composite superstructures of TiO_2@C anode, and $LiFePO_4$@C cathode. To explore the oxidation states of metal and non-metal globule components of super-scale models of TiO_2@C// $LiFePO_4$@C (anode//cathode) compositions, we carried out extensive surface sensitive information on the VST(i)@C and FRTO@C samples using continuously irradiation to X-ray beam during XPS analyses as shown in Figs. S12-A(a-e) and B(a-c).

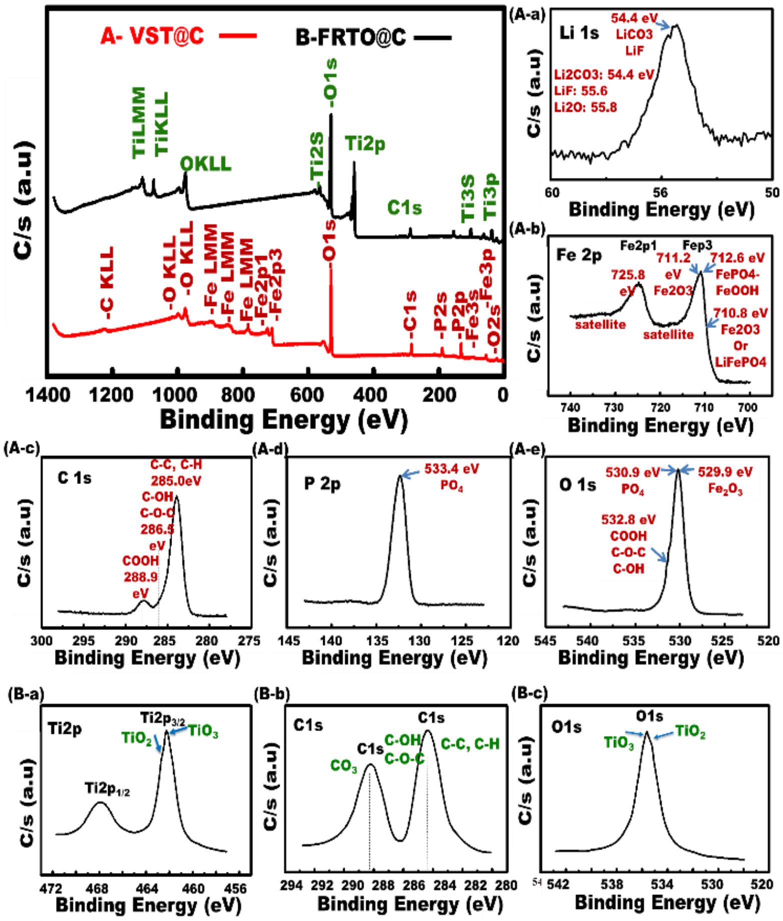

Fig. S12 A XPS survey spectrum of the prepared VST(i)@C. **B** XPS survey spectrum of the prepared FRTO@C

Figure S12-A illustrates the XPS peaks at 711.2 and 530.9 eV are the binding energies (Bes) related to Fe 2p and O 1s, respectively. The Fe-BE domain is markedly related to its oxidation state (Fe^{2+}). Some other oxidation states of Fe-domains couldn't be observed via XPS analysis. In addition, the P 2p, Li 1s, and C 1s peaks are shown at 133.4, 55.8, and 284.9 eV, respectively, attributable to the formation of orthorhombic olivine-type $LiFePO_4$ structure with homogenous molecular phase formation along $LiFePO_4$@C cathode surfaces.

The XPS analysis of FRTO@C was carried out and surface states of the samples were revealed, as shown in Fig. S12B. XPS peaks show evidence of the existence of C 1s, O 1s, and Ti 2p surface states with formation of super-scale TiO_2@C anode or FTRO@C hierarchy.

The binding energy of Ti 2p exhibits two peaks at 468 and 462 eV which are related to present of Ti 2p1/2 and Ti 2p3/2 stated. The observed peak at 534 eV can be attributed to O 1s state. The overall peaks of Ti 2p1/2, Ti 2p3/2, and O 1s indicate the formulation of TiO_2 phases. Finally, C 1s surface state exhibits two peaks at 288.5 and 284.2 eV owing to sp^2 C=O and C-C/C-O bonds, indication the successful formation of homogeneous rutile TiO2 phase in multi-composite TiO_2@C anode phase.

Together, both the XPS and XRD results ensured that VST@C and FRTO@C composites are consisted of anisotropic multi-reactive components oriented in crystal structures of olivine $LiFePO_4$@C (VST@C) and rutile TiO_2@C (FRTO@C); respectively. The multi-functional surface composites are probably responsible for the efficient electrochemical performance of both electrodes in design of power hierarchy LIBs built-up sets for maintaining high Li^+ ion-diffusivity, facile electron transfer, and excellent charge–discharge rates, high-energy- density with long-period of timescale reusability of LIBs.

S15 Electrochemical Performance of Half-cell FRTO@C Anode

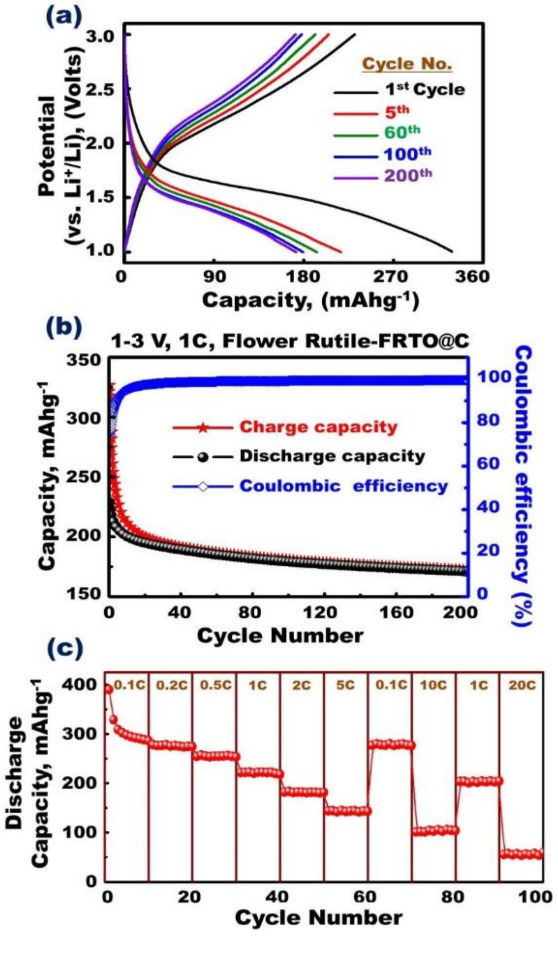

Fig. S13 a The charge-discharge voltage profiles of first and multi-cycles up to 200 cycles-at current rate 1C of half-cell FRTO@C anode (2032 coin-type half-cell tests with a Li counter electrode). **b** Long term cycling performance (stability) and Coulombic efficiency for half-cell FRTO@C, at rate of 1C up to 200 cycles, and in voltage range 1-3V at room temperature. **c** Rate capability performance over a range of 1–3V among half-cell FRTO@C anode at various current rates from 0.1 to 20 C. All electrochemical measurements for FRTO@C half-cell anode were operated at room temperature.

S16 Influence of Super-Hierarchal Shaped VST Cathodes on LIB Performance

To explore the effect of uniformly-ordered LFPO@C cathode that has morphologically vertical star tower VST@C structures and designated with flexible, multiple building blocks and units at high-end tower roofs on the LIB performance, we studied the non-uniform LFPO@C cathode in the electrochemical reaction of half-cell cathodes. First, we have fabricated non-uniformly morphological $LiFePO_4$ cathode via simple hydrothermal treatment, as follows:

A mixture of Lithium acetate dihydrate ($CH_3COOLi·2H_2O$), iron III nitrate nonhydrate ($Fe(NO_3)_3·9H_2O$), phosphoric acid (H_3PO_4) and Et/EG mixture ratios (100%:0) was inserted one-pot in time-independent treatment process at 170 °C for 12 h. The non-uniform or not-controlled LFPO materials were calcined in a muffle furnace under Ar at 450 °C for 6 h. In addition the not-controlled LFPO@C materials and LFPO@C electrode were fabricated according the typical procedures used for VST@ cathode electrodes, respectively, see the experimental section. FE-SEM micrographs show non-uniformly LFPO@C morphological structures, as seen in Fig. S14a, b.

To study the effect of cathode structure ordering and hierarchy on the electrochemical performance of half-cell cathode LIBs, we used non-controlled $LiFePO_4$@C cathode morphology and VST-(i)@C hierarchy half-cell cathode LIB models, as shown in Fig. S14c. Figure S14c presented the typical 1st cycle charge-discharge voltage profiles of structurally not-controlled $LiFePO_4$@C and hierarchy controlled VST-(i)@C half-cell cathodes at different current rates 0.1 C, 1 C, and 10 C designated in 2032 coin-type half-cell tests with a Li counter electrode between 2.0 and 4.3 V. The VST-(i)@C exhibits an excellent discharge capacity at C-rates ranging from 0.1 to 10 C compared with the structurally non-controlled $LiFePO_4$@C half-cells. The superior long-term cycling performance and stability of the current VST(i)@C cathodes are presented in Fig. S14d. VST-(i)@C retains 99.5% of its 1st-cycle capacity after 100 cycles at 0.1 C. Meanwhile, non-controlled structure $LiFePO_4$@C retains 75% of its initial capacities after 100 cycles at 0.1 C, respectively. The VST-(i)@C half-cell cathode does not demonstrate any capacity fading over 100 cycles at a rate of 0.1 C, which indicates the hotkeys of the open-star tower VST-(i)@C hierarchy in high electrochemical reversibility during the lithiation/delithiation process. The hierarchal directions of VST-(i)@C cathode in the lateral, vertical (axial), and longitudinal axes, offering bowl-shaped ridges/alcoves, extensive convex-up planar needles arranged in a tip-to-tip configuration, and carved trenches in the flanks result in the predominance of high-mobility electron/ion flows and the enhancement of surface potential compared with the non-uniform $LiFePO_4$@C cathode (Fig. S14).

The rate capability of VST(i)@C (controlled) and generally non-uniform $LiFePO_4$@C structure cathodes was evaluated at different rates (0.1, 0.2, 0.5, 1, 2, and 5 C then back to 0.1 C and 10 C and then back to 1 and 20 C, with 10 cycles at each rate at room temperature (Fig. S14e). The reversible discharge capacity of controlled VST@C and non-controlled LFPO@C cathodes has retained 66 and 8.4% of its initial capacities, respectively, at a rate of 20 C over 100 cycles. In other words, the specific capacity usually decreases as the C-rate increases for all tested cathodes. VST-(i)@C cathode shows a superior rate performance at a rate of 20 C and after 100 cycles while poor rate capability observed for non-controlled LFPO@C structure. The excellent electrochemical performance, high capacity at high rate capability, and long cycle life of VST-(i)@C cathode may be ascribed to its hierarchy regular morphology and 3D superstructure composite with single, upper-top-capped pyramidal prism

headings at the end-zone incidents of the multi-directional wing scales. The capping prism-base shapes have clear apex, edge, and vertex-ended surfaces, which play important roles in achieving a high volumetric energy density, excellent rate capability, and Li-ion diffusion throughout all dimensions and loops of the VST-(i)@C cathode.

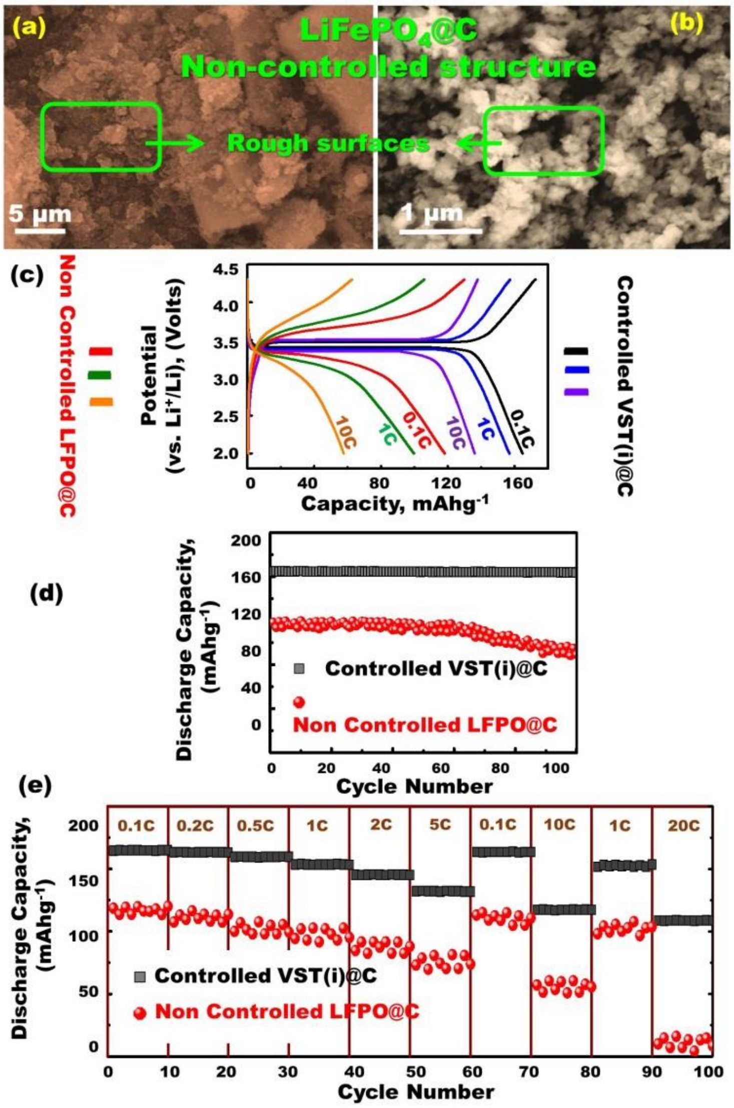

Fig. S14 a, b Low and high-resolution FE-SEM images of $LiFePO_4$@C cathode material without control the structure. **c** The first cycle charge-discharge voltage profiles of structurally non-controlled $LiFePO_4$@C and controlled hierarchy VST-(i)@C half-cell cathodes at different current rates 0.1 C, 1 C, and 10 C designated in (2032 coin-type half-cell tests with a Li counter electrode). **d** Cycling performance (stability) for structurally non-controlled $LiFePO_4$@C and hierarchally-controlled VST-(i)@C half-cell cathodes, at rate of 0.1 C for 100 cycles. **e** Rate capability performance for structurally non-controlled $LiFePO_4$@C and hierarchally-controlled VST-(i)@C half-cell cathodes from 0.1 to 20 C. All electrochemical measurements were operated a within voltage range of 2.0– 4.3 V, at room temperature.

In this regard, Fig. S14c-e confirmed the remarkable advantages of VST-(i)@C, leading to its superb retention performance, excellent rate capability, and high cycling stability. The superior choice of VST-(i)@C-cathode architectonics merits the following:

(i) Rapid electron movement and Li+-diffusion kinetics at interfaces during the lithiation/delithiation process;
(ii) Excellent electronic contact, and high electronic conductivity, as well as its ability to reduce the Li^{+}–ions diffusion path, and to facilitate the transport of electrons;
(iii) 3D hierarchical super-scale building blocks with multi-diffusive meso/macro open sites;
(iv) Open-multi-direction, scalable mosaic towers, which comprise a mass of curved, prismed room grooves;
(v) Corridor tunnels and evoke a crop of bowels, with each kernel cavity representing individual cave spaces for facilitating diffusion mobility and ensuring the accommodation of Li+ ions during charge/discharge process.

Given its outstanding electrochemical performance, VST-(i)@C is considered as one of potential candidates for meeting the high-power and high-energy requirements of LIBs and EV applications.

S17 Effect of C-dot Well-dressing and Content on Capacity and Cycling Stability of VST(i) Cathode

Various electrochemical experiments of VST-(i) and VST-(i)@C half-cell cathodes have been carried out to confirm the effect of well-ordered decoration and sustainable coating of C-shell dressers along superhierarchally-shaped VST cathodes in improving the kinetics of electron/Li^{+} ion transportation during lithiation/delithiation Fig. S15. Figure S15a shows the charge/discharge cycling of the VST(i) and VST(i)@C cathodes. The built-in half-cell cathodes practically charged to 4.3 V at 0.1 C, maintained at 4.3 V for 1 h, and discharged to 2.0 V at 0.1 C. The finding indicated that the presence of well-dressing C-dot enhanced the electrochemical performance. Evidentially, the super-hierarchal VST@C cathode displayed higher storage capacity than that of the VST cathode. At the first cycle (0.1 C), the VST-(i)@C shows discharge capacity (165 mAh g^{-1}), which is higher than that of VST (153.2 mAh g^{-1}) (Fig. S15 a). Figure S15b shows superior capacity retention at the long-term cyclability (i.e., stability) of the VST@C-cathode, indicating that the 99.5% capacity was retained of the initial capacity after 100 cycles for the VST@C cathode (0.1 C). However, the VST cathode maintained 94% of its initial capacity. The low charge transfer resistance of the VST@C cathode may be due to high electronic conductivity and excellent electron/ion transfer kinetics during lithiation/delithiation. The continuous distribution of C-shell dressing of the few nanometres ($\leq$ 5 nm) C-dot bump maps along the super hierarchal VST cathode significantly enhanced Li^{+}-ion diffusion coefficient (Figs. 1b, 1c, and 1k). The VST@C electrode exhibits high Li-storage capacity and excellent rate performance. The well-dressing of C-dot bump maps along active material surface ingredients may create multi-pool bowls for reactive and contiguous surface coverage of electron/Li ions during discharge/charge cycles.

Together, the dressing of C-dot bump maps along the super-hierarchal anode/cathode electrodes did not change in the atomic-scale ordering structures or disturb in atomic localizations. The C-dot shelled VST-cathode may provide:

(i) 3D affordable outer surface ridges and bowls;
(ii) Reduction of the binding energy surface;
(iii) Enhancement of the electronic surface transport functionality, and electrical conductivity of electrode surfaces;
(iv) Retention of electron/Li^{+} ion pathways along the surface coverage without vortex surface hindrance that may be created due to the mechanical shock and atomic-scale volume expansion in single-crystal super-structures associated with frequent and continuous lithiation/delithiation cycling;

(v) Protection of the electrode cuticles and building block hierarchy from degradation against the heating out created within multiple charge/discharge cycles.

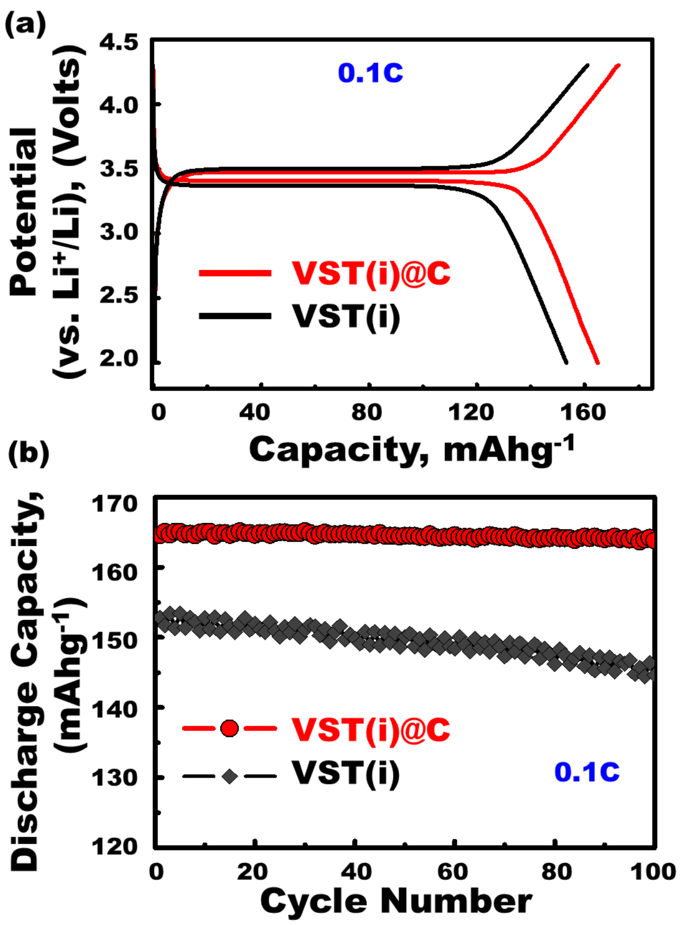

Fig. S15 a The charge-discharge voltage profiles at a current rate 0.1C of half-cell VST(i) and VST(i)@C cathodes. **b** Cycling performance (stability) for VST(i) and VST(i)@C cathodes in half cell 2032-coin cell with Li foil as counter electrode, at rate of 0.1 C for 100 cycles

S18 Calculating Processes of Specific Energy Density of VST-(i)@C//FRTO@C (Cathode//Anode) Full-scale LIB-model

As shown in Table S1, the mass fraction of a VST-(i)@C cathode active material in a pouch LIB cell design is approximately 44%. Then, the practical value of a specific energy density for the (LiFePO4//TiO_2) VST-(i)@C//FRTO@C super-hierarchical building blocks full-scale LIB was found to be 127 Wh kg^{-1}. Moreover, the theoretical capacity ($Q_{theoretical}$) of a LIB cell that has been calculated by Faraday's law represented that the Q of $LiFePO_4$ and TiO_2 was found to be =170 and 335 mAh g^{-1}, respectively [S1-S4]. The idealized capacity ratio of $LiFePO_4$: TiO_2 = (170 Ah kg^{-1}/335 Ah kg^{-1}) = 0.51. Table S1 shows that ~34% weight allowance for both electrode balances is considered for a practical pouch Li-ion cell components. Thus, the full scale LIB with a specific capacity of ((170 Ah)/(1kg +0.51 kg))/1.34 is about 84.0 Ah kg^{-1}. One can then calculate the specific energy density (i.e., theoretically) for the full-scale $LiFePO_4$//TiO_2 battery to be (1.6 V × 84 Ah kg^{-1}) equal 134 Wh kg^{-1}. The results confirmed that the specific energy density for the VST-(i)@C//FRTO@C super-hierarchical building blocks full-scale LIB has a value of 127 Wh kg^{-1} (i.e., practically) and 134 Wh kg^{-1} (i.e., theoretically). The low value of the practical capacity compared with its theoretical value may be due to the following:

1. All the Li^+-ions can't be removed from the lattice of the host cathode/anode material after lithiation/delithiation process;
2. The rest-loaded Li^+ along electrode coverage surfaces needs high voltage, which is mainly above the cut-off potential (i.e., it is not applicable in normal designated conditions).

Together, either theoretically or practically, the specific energy density of VST-(i)@C//FRTO@C super-hierarchical building blocks full-scale LIB offers more salutary limit than that of driving range requirement for EVs. The proposed battery system based on VST-(i)@C//FRTO@C super-hierarchical building blocks integrates the state-of-the-art developments in Li-battery technology.

S19 Optimization of Full Cell (Balancing) – (N/P) Ratio

The effect of N/P ratio (N = negative anode electrode capacity; and P = positive cathode electrode capacity in mAh) on electrochemical reaction and full cell performance was studied. However, due to the differences in reversible specific capacities of negative and positive electrode, a suitable control of mass loading of both negative and positive electrodes is necessary to realize equal discharge specific capacity for both electrodes during LIB-cell operation [S6- S9]. Furthermore, to avoid risk of lithium metal plating, which is considered as a severe aging and safety-deteriorating process [S10, S11], a slight oversizing of the capacity of negative electrodes ($(N{:}P)_{Cap}$ capacity ratio >>1–1.2: 1) is additionally required for better safety and battery life, termed as "capacity balancing" or simply "balancing" in literature [S12- S17].

To optimize a full cell (balancing) – (N/P) ratio, we apply electrochemical cell design of 3D super-scalable full-scale $LiFePO_4$//TiO_2 cathode//anode stacked layers pouch LIB-model. Thus, we rationally control the optimal trade–off between better safety (oversizing of negative electrode capacity, $(N{:}P)_{Cap}$ capacity ratio = >1:1) and optimum specific energy (equal capacities of negative and positive electrode, $(N{:}P)_{Cap}$ capacity ratio =1:1). In this study, we found that our proposed 3D super-scalable full-scale $LiFePO_4$//TiO_2 cathode//anode stacked layers pouch LIB-model is optimized mass loading with $(N{:}P)_{Cap}$ capacity ratio ≈ 1.02 – 1.1:1.

We built the full-scale stacking sequence of VST-(i)@C//FRTO@C (cathode//anode) electrodes with dimensions of 35 mm (width), 55 mm (length) and ~2- 2.5mm (thickness). The weight fraction calculations of these stacked pouch cell components show that the active mass of cathode and anode are 1.96 and 0.99 g, respectively. The VST-(i)@C//FRTO@C (cathode//anode) stacked pouch LIB-model was designed used 6 layers of cathode with 10 sides loaded Al-foil (10 µm) positive collector, and 5 layers of anodes with 10 sides loaded Cu-foil (8 µm) negative collector. In order to optimize the full-scalable cell, the total electrode area of the cathode and anode in the full-cell are 150 and 143 cm^2, respectively.

S20 Estimation of Volumetric Energy Density

On the base of electrochemical cells of stacked layers pouch full-LIB-scale built-up set and the superior choice anode/cathode architectonics LIB-models, the volumetric energy density was found to be 172 Wh L^{-1}. In turn, the stored energy density was found to be 6.1 Wh and its volumetric energy density ~ 381 Wh L^{-1}; which is reasonable value comparing to the range of the volumetric energy density for most commercial 18650-LIBs 250–500 Wh L^{-1}, as produced in market trade names of Panasonic NCR18650B, and NCR18650GA for EVs [S18-S20]. Accordingly, the full cell 18,650 cylindrical design gives a real space of evidence to be used as a model for our proposed VST-(i)@C//FRTO@C material-based electrodes.

S21 Electrochemical Impedance Spectroscopy

The EIS-experiments of the structurally non-controlled $LiFePO_4$@C sample and controlled VST(i)-non-coated and VST(i-vii)@C-coated cathode samples were examined, see Fig. S16 and Table S2.

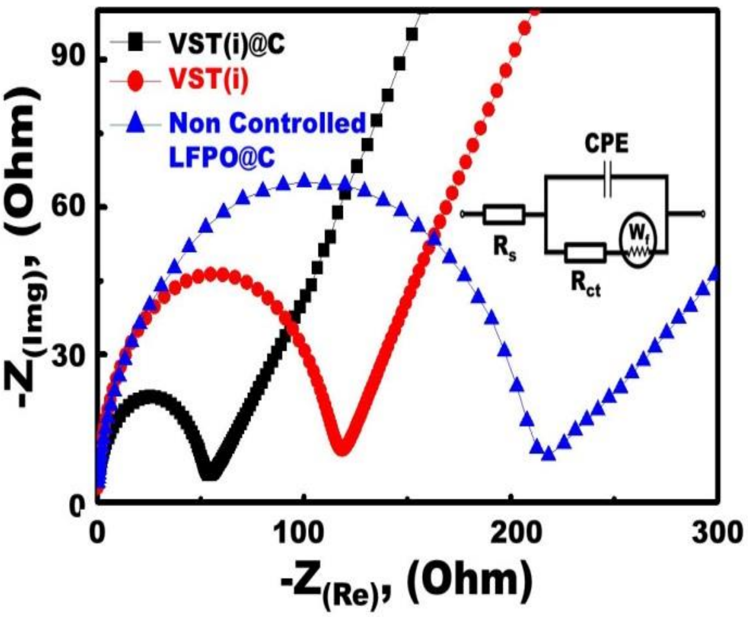

Fig. S16 Nyquist plots at room temperature of the general structurally not-controlled $LiFePO_4$ sample and controlled VST(i)-not-coated and VST(i-vii)@C-coated samples by ~ 5 nm C-particles. The inset in figure is the simple equivalent circuit used to fit the impedance data.

Table S2 Electrode kinetic parameters of $LiFePO_4$ composites obtained from equivalent circuit fitting of electrochemical impedance spectroscopy EIS-experimental data

Sample	R_s (Ω)	R_{ct} (Ω)	I_0 (mA)
LiFePO4 (Non-controlled Structure)	1.9	213	0.1206
VST(i)	1.6	118.4	0.217
VST(i)@C	1.3	54.5	0.47
VST(ii)@C	1.6	86.4	0.297
VST(iii)@C	1.5	104.5	0.246
VST(iv)@C	1.7	120	0.214
VST(v)@C	1.5	137	0.187
VST(vi)@C	1.3	150.4	0.171
VST(vii)@C	1.8	187	0.137

Table S2 indicated that the VST(i)@C composite has minimum R_{ct} and higher exchange current density (I_0) than that of other cathode samples. The key facts are that hierarchally-controlled structure and carbon coating effect play a role in the performance of half-cell cathodes. For instance, the hierarchally-controlled VST-(i)@C cathode structure is connected with four lateral/longitudinal exposure wings or vestibule corridors, offering vast active surfaces of axially, laterally, longitudinally and horizontally directional movements of ions/electrons. This structural feature leads to excellent electron/ion transfer kinetics of the VST-(i)@C cathode through the lithiation/delithiation process.

S22 A Comparison Between the VST-(i)@C Half-scale LIB and the Other Reported $LiFePO_4$ Cathodes

Table S3 A comparison between the VST-(i)@C half-scale LIB and the other reported $LiFePO_4$ cathodes

Cathode material	Nominal Voltage (V)	Cycles	Specific Capacity (mAh g^{-1})	Coulombic efficiency	Refs.
$LiFePO_4$	3.4	retained ~100% of its reversible capacity after 100 cycles	155 At 1 C	Not mentioned	**[S21]**
$LiFePO_4$	3.45	retained ~100% of its reversible capacity after 120 cycles	79 at 1 C	Not mentioned	**[S22]**
$LiFePO_4$	3.4	95% of its initial capacity after 100 cycles at 10 C	148	Not mentioned	**[S23]**
$LiFePO_4$	3.4	99% of its initial capacity 100 mAh/g after 1000 cycles at 20C	100	~ 100%	**[S24]**
$LiFePO_4$	3.4	Not mentioned	119	Not mentioned	**[S25]**
$LiFePO_4$	3.4	100 % of its initial capacity after 100 cycles at 1 C	140	~100%	**[S26]**
$LiFePO_4$	3.4	100 % of its initial capacity after 100 cycles at 1 C	152	~100%	**[S27]**
$LiFePO_4$@C (AF@C)	3.45	Retains ~100% after 100 cycles	158.8	~100%	**Current Work**

S23 A comparison Between the FRTO@C Half-scale LIB and the Other Reported TiO_2 Anodes

Table S4 A comparison between the FRTO@C half-scale LIB and the other reported TiO_2 anodes

**Anode material**	**Nominal Voltage (V)**	**Cycles**	**Specific Capacity (mAh g^{-1})**	**Coulombic efficiency**	**Refs.**
Rutile TiO_2	1.7	retained 50% of its reversible capacity after 100 cycles	250 At 0.1 C	~ 100% after 100 cycles	**[S28]**
Rutile TiO_2	1.5	retained 77% of its reversible capacity after 40 cycles	223 at 0.1 C	Not mentioned after 100 cycles	**[S29]**
Anatase TiO_2	1.73	76% of its initial capacity after 30 cycles at 0.2 C	~ 300	Not mentioned	**[S30]**
P25, a commercial titania powder from Degussa	1.7	61% of its initial capacity after 30 cycles at 0.2 C	95	Not mentioned	**[S30]**
TiO_2	1.74	70% of its initial capacity after 100 cycles at 0.2C	270	Not mentioned	**[S31]**
TiO_2	0.8	55% of its initial capacity after 30 cycles	310	~100%	**[S32]**
TiO_2@C (FRTO@C)	1.7	54% of its initial capacity after 200 cycles at 1 C	329	~100%	**Current Work**

S24 A Comparison Between the VST-(i)@C//FRTO@C Full-cell LIB Electrode system and the Other Reported $LiFePO_4$//TiO_2 Full Cells

Table S5 A comparison between the VST-(i)@C//FRTO@C full-cell LIB electrode system and the other reported $LiFePO_4$//TiO_2 full cells

Cathode material	Anode material	Nominal Voltage (V)	Cycles	Specific Capacity mAh g^{-1}	Coulombic efficiency	Refs
$LiFePO_4$	Anatase TiO_2 hollow nanofibers	1.4	retained 88% of its reversible capacity after 300 cycles	103	> 99 %	[S33]
$LiFePO_4$	Rutile TiO_2	1.8	retained 50% of its reversible capacity after 40 cycles	150	Not mentioned	[S34]
$LiFePO_4$	Anatase TiO_2	1.6	81% of its initial capacity after 300 cycles at 20 C	160	Not mentioned	[S35]
$LiFePO_4$	anatase/graphene	1.6	700	127	~ 100%	[S36]
LiFePO4	spinel $Li_4Ti_5O_{12}$/C	1.8	Retain 98.1% after 400 cycles	167	~100%	[S37]
$LiFePO_4$	spinel Li4Ti_5O_{12}	1.65	Retain 98.9% after 100 cycles	150	~100%	[S38]
$LiFePO_4$@C (VST(i)@C)	TiO_2@C (FRTO@C)	1.8	Retains 94.2% after 2000 cycles at 1 C	160	~100%	Current Work

Supplementary References

[S1] A.W. Golubkov, D. Fuchs, J. Wagner, H. Wiltsche, C. Stangl et al., Thermal-runaway experiments on consumer Li-ion batteries with metal-oxide and olivin-type cathodes. RSC Adv. **4**, 3633-3642 (2014). https://doi.org/10.1039/C3RA45748F

[S2] S. Liu, J. Yu, M. Jaroniec, Anatase TiO_2 with dominant high-energy {001} facets: synthesis, properties, and applications. Chem. Mater. **23**, 4085–4093 (2011). https://doi.org/10.1021/cm200597m

[S3] J.S. Chen, L.A. Lou, X.W.Lou, SnO_2 hollow structures and TiO_2 nanosheets for lithium-ion batteries. J. Mater. Chem. **21**, 9912–9924 (2011). https://doi.org/10.1039/c0jm04163g

[S4] L. Zuniga, V. Agubra, D. Flores, H. Campos, J. Villareal, M. Alcoutlabi, Multichannel hollow structure for improved electrochemical performance of TiO_2/carbon composite nanofibers as anodes for lithium ion batteries. J. Alloys Compd. **686**, 733–743 (2016). https://doi.org/10.1016/j.jallcom.2016.06.089

[S5] X. Yang, D. Teng, B. Liu, Y. Yu, X. Yang, Nanosized anatase titanium dioxide loaded porous carbon nanofiber webs as anode materials for lithium-ion batteries. Electrochem. Commun. **13**, 1098-1101 (2011). https://doi.org/10.1016/j.elecom.2011.07.007

[S6] O.K. Park, Y. Cho, S. Lee, H.C. Yoo, H.K. Song, J. Cho, Who will drive electric vehicles, olivine or spinel? Energy Environ. Sci. **4**, 1621–1633(2011). https://doi.org/10.1039/c0ee00559b

[S7] V. Srinivasan, J. Newman, Design and optimization of a natural graphite/iron phosphate lithium-ion cell. J. Electrochem. Soc. **151**, A1530–A1538 (2004). https://doi.org/10.1149/1.1785013

[S8] H.M. Wu, I. Belharouak, H. Deng, A. Abouimrane, Y.K. Sun, K. Amine, Development of $LiNi_{0.5}Mn_{1.5}O_4$ / $Li_4Ti_5O_{12}$ system with long cycle life. J. Electrochem. Soc. **156**, A1047–A1050 (2009). https://doi.org/10.1149/1.3240197

[S9] K. Eberman, P.M. Gomadam, G. Jain, E. Scott, Battery/energy technology (general) - 216th Ecs Meeting. **25** , 47–58 (2010).

[S10] J. Vetter, P. Novak, M.R.Wagner, C. Veit, K.C. Moller et al., Ageing mechanisms in lithium-ion batteries. J. Power Sources **147**, 269 (2005). https://doi.org/10.1016/j.jpowsour.2005.01.006

[S11] H.Y. Zheng, L. Tan, L. Zhang, Q.T. Qu, Z.M. Wan, Y. Wang, M. Shen, H.H. Zheng, Correlation between lithium deposition on graphite electrode and the capacity loss for $LiFePO_4$/graphite cells. Electrochim. Acta **173**, 323 (2015). https://doi.org/10.1016/j.electacta.2015.05.039

[S12] S. Krueger, R. Kloepsch, J. Li, S. Nowak, S. Passerini, M.Winter, How do reactions at the anode/electrolyte interface determine the cathode performance in lithium-ion batteries? J. Electrochem. Soc. **160**, A542 (2013). https://doi.org/10.1149/2.022304jes

[S13] C.S. Kim, K.M. Jeong, K. Kim, C.W. Yi, Effects of capacity ratios between anode and cathode on electrochemical properties for lithium polymer batteries. Electrochim. Acta **155**, 431 (2015). https://doi.org/10.1016/j.electacta.2014.12.005

[S14] K. Kleiner, P. Jakes, S. Scharner, V. Liebau, and H. Ehrenberg, Changes of the balancing between anode and cathode due to fatigue in commercial lithium-ion cells. J. Power Sources. **317**, 25 (2016). https://doi.org/10.1016/j.jpowsour.2016.03.049

[S15] P. Meister, H.P. Jia, J. Li, R. Kloepsch, M. Winter, T. Placke, Best practice: performance and cost evaluation of lithium ion battery active materials with special

emphasis on energy efficiency. Chem. Mat. **28**, 7203 (2016). https://doi.org/10.1021/acs.chemmater.6b02895

[S16] P. Arora, R.E. White, M. Doyle, Capacity fade mechanisms and side reactions in lithium - ion batteries. J. Electrochem. Soc. **145**, 3647 (1998). https://doi.org/10.1149/1.1838857

[S17] D. Andre, S.J. Kim, P. Lamp, S.F. Lux, F. Maglia, O. Paschos, B. Stiaszny, Future generations of cathode materials: an automotive industry perspective. J. Mater. Chem. A **3**, 6709 (2015). https://doi.org/10.1039/C5TA00361J

[S18] https://en.wikipedia.org/wiki/Lithium-ion_battery

[S19] "Lithium Ion NCR18650B" (PDF). Panasonic. Retrieved 7 October 2016. https://www.batteryspace.com/prod-specs/NCR18650B.pdf

[S20] "ncr18650ga datasheet specs for Panasonic Sanyo 18650 battery- NCR18650GA" (PDF). Retrieved 2 July 2017. https://www.orbtronic.com/content/Datasheet-specs-Sanyo-Panasonic-NCR18650GA-3500mah.pdf

[S21] K. Muxina, T. Izumi, Synthesis of carbon-coated $LiFePO_4$ nanoparticles with high rate performance in lithium secondary batteries. J. Power Sources **195**, 3661-3667 (2010). https://doi.org/10.1016/j.jpowsour.2009.11.147

[S22] L.Yu, D. Cai, H. Wang, M.-M. Titirici, Synthesis of microspherical $LiFePO_4$-carbon composites for lithium-ion batteries. Nanomaterials **3(3)**, 443-452 (2013) https://doi.org/10.3390/nano3030443

[S23] X. Loua, Y. Zhang, Synthesis of $LiFePO_4$/C cathode materials with both high-rate capability and high tap density for lithium-ion batteries. J. Mater. Chem. **21**, 4156-4160 (2011). https://doi.org/10.1039/c0jm03331f

[S24] B. Guo, H. Ruan, C. Zheng, H. Fei, M. Wei, Hierarchical $LiFePO_4$ with a controllable growth of the (010) facet for lithium-ion batteries. Sci. Rep. **3**, 2788 (2013). https://doi.org/10.1038/srep02788

[S25] J. Wanga, X. Sun, Understanding and recent development of carbon coating on $LiFePO_4$ cathode materials for lithium-ion batteries. Energy Environ. Sci. **5**, 5163-5185 (2012). https://doi.org/10.1039/C1EE01263K

[S26] J. Zhang, J. Lu, D. Bian, Z. Yang, Q. Wu, W. Zhang, Solvothermal synthesis of hierarchical $LiFePO_4$ microplates with exposed (010) faces as cathode materials for lithium ion batteries. Ind. Eng. Chem. Res. **53(31)**, 12209-12215 (2014). https://doi.org/10.1021/ie501743b

[S27] H. Liu, C. Miao, Y. Meng, Y.-B. He, Q. Xu, X. Zhang, Z. Tang, Optimized synthesis of nano-sized $LiFePO_4$/C particles with excellent rate capability for lithium ion batteries. Electrochim. Acta **130**, 322-328 (2014). https://doi.org/10.1016/j.electacta.2014.03.034

[S28] T. Tao, Y. Chen, Direct synthesis of rutile TiO_2 nanorods with improved electrochemical lithium ion storage properties. Mater. Lett. **98**, 112-115, (2013). https://doi.org/10.1016/j.matlet.2013.01.132

[S29] H. Qiao, Q. Luo, Q. Wei, Y. Cai, F. Huang, Electrochemical properties of rutile TiO_2 nanorods as anode material for lithium-ion batteries. Ionics **18**(7), 667–672 (2012). https://doi.org/10.1007/s11581-012-0672-5

[S30] K. Saravanan,K. Ananthanarayanan, P.Balay, Mesoporous TiO_2 with high packing density for superior lithium storage. Energy Environ. Sci. **3**, 939-948 (2010). https://doi.org/10.1039/c003630g

[S31] J. Liang, Z. Wang, Z. Li, X. Wang, K. Yu, Fabrication of nanostructured TiO_2 using a solvothermal reaction for lithium-ion batteries. Nanomater. Nanotech. **6** , 151 (2016). https://doi.org/10.5772/62420

[S32] Z. Hong, K. Zhou, Z. Huang, M. Wei, Iso-oriented anatase TiO_2 mesocages as a high performance anode material for sodium-ion storage. Sci. Rep. **5**, 11960 (2015). https://doi.org/10.1038/srep11960

[S33] X. Zhang, V. Aravindan, P. Suresh Kumar, H. Liu, J. Sundaramurthy, S. Ramakrishna, S. Madhavi, Synthesis of TiO_2 hollow nanofibers by co-axial electrospinning and its superior lithium storage capability in full-cell assembly with olivine phosphate. Nanoscale **5**, 5973-5980 (2013). https://doi.org/10.1039/c3nr01128c

[S34] J. Hassoun, M. Pfanzelt, P. Kubiak, M. Wohlfahrt-Mehrens, B. Scrosati, An advanced configuration TiO_2/$LiFePO_4$ polymer lithium ion battery. J. Power Sources **217**, 459-463 (2012). https://doi.org/10.1016/j.jpowsour.2012.05.071

[S35] Z. Guo, X. Dong, D. Zhou, Y. Du, Y. Wang, Y. Xia, TiO_2(B) nanofiber bundles as a high performance anode for a Li-ion battery. RSC Adv. **3**, 3352-3358 (2013). https://doi.org/10.1039/c2ra23336c

[S36] D. Choi, D. Wang, V.V. Viswanathan, I.T. Bae, W. Wang et al., Li-ion batteries from $LiFePO_4$ cathode and anatase/graphene composite anode for stationary energy storage. Electrochem. Commun. **12(3)**, 378-381 (2010). https://doi.org/10.1016/j.elecom.2009.12.039

[S37] C-C.Yang, H-C. Hu, S.J. Lin, W-C. Chien, Electrochemical performance of V-doped spinel $Li_4Ti_5O_{12}$/C composite anode in Li-half and $Li_4Ti_5O_{12}$/$LiFePO_4$-full cell. J. Power Sources **258**, 424-433 (2014). https://doi.org/10.1016/j.jpowsour.2014.01.130

[S38] J. Morales, R. Trocoli, S. Franger, J. Santos-Pena, Cycling-induced stress in lithium ion negative electrodes: LiAl/$LiFePO_4$ and $Li_4Ti_5O_{12}$/$LiFePO_4$ cells, Electrochim. Acta **55** , 3075e3082 (2010). https://doi.org/10.1016/j.electacta.2009.12.104
